# Identification of novel transcripts and peptides in developing murine lens

**DOI:** 10.1038/s41598-018-28727-w

**Published:** 2018-07-24

**Authors:** Shahid Y. Khan, Muhammad Ali, Firoz Kabir, Ruiqiang Chen, Chan Hyun Na, Mei-Chong W. Lee, Nader Pourmand, Sean F. Hackett, S. Amer Riazuddin

**Affiliations:** 1 The Wilmer Eye Institute, Johns Hopkins University School of Medicine, Baltimore, MD 21287 USA; 2 Department of Biological Chemistry, Johns Hopkins University School of Medicine, Baltimore, MD 21205 USA; 3 Department of Biomolecular Engineering, University of California, Santa Cruz, CA 94305 USA

## Abstract

We previously investigated the transcriptome and proteome profiles of the murine ocular lens at six developmental time points including two embryonic (E15 and E18) and four postnatal time points (P0, P3, P6, and P9). Here, we extend our analyses to identify novel transcripts and peptides in developing  mouse lens. We identified a total of 9,707 novel transcripts and 325 novel fusion genes in developing mouse lens. Additionally, we identified 13,281 novel alternative splicing (AS) events in mouse lens including 6,990 exon skipping (ES), 2,447 alternative 3′ splice site (A3SS), 1,900 alternative 5′ splice site (A5SS), 1,771 mutually exclusive exons (MXE), and 173 intron retention (IR). Finally, we integrated our OMIC (Transcriptome and Proteome) datasets identifying 20 novel peptides in mouse lens. All 20 peptides were validated through matching MS/MS spectra of synthetic peptides. To the best of our knowledge, this is the first report integrating OMIC datasets to identify novel peptides in developing murine lens.

## Introduction

Next-generation RNA sequencing (RNA-Seq) has significantly enhanced our ability to decipher whole transcriptomes through the gene expression quantification, identification of novel transcripts, detection of fusion genes, and isoform diversity^1–7^. The mouse genome encodes 53,715 genes, including 21,981 protein-coding genes (GENCODE Ver. M17). However, the total number of transcripts encoded by these genes is believed to be much higher suggesting multiple layers of complexity at the transcriptome level^8,9^.

Fusion genes describe a phenomenon of hybrid RNA resulting from read-through transcripts, composed of two different genes formed during chromosomal re-arrangements^10,11^. Fusion genes could be a product of cis-splicing as well as trans-splicing^12–15^. Alternative mRNA splicing, a phenomenon more prevalent in higher eukaryotes, provides additional diversity in gene expression^8,16^, and according to some estimates >95% of human multi-exonic mRNAs undergo mRNA splicing^9,17,18^.

The ocular tissue especially the retina has been characterized extensively using multiple next-generation based transcriptome studies that revealed highly diverse annotated and novel transcriptome and novel isoforms^19,20^. In contrast, the characterization of the lens expression profile has received less attention and fewer next-generation RNA sequencing-based studies have been completed. Recently, Srivastava and colleagues identified novel transcripts and splicing alterations in developing murine lens^21^.

We previously reported the mouse lens coding and non-coding transcriptome at six developmental time points including two embryonic (E15 and E18) and four postnatal stages (P0, P3, P6, and P9)^22,23^. More recently, we reported a comprehensive proteome of the mouse lens at the same six developmental time points^24^. Here, we extend our analyses to identify novel transcripts and peptides in developing mouse lens.

## Results

**Figure 1:**
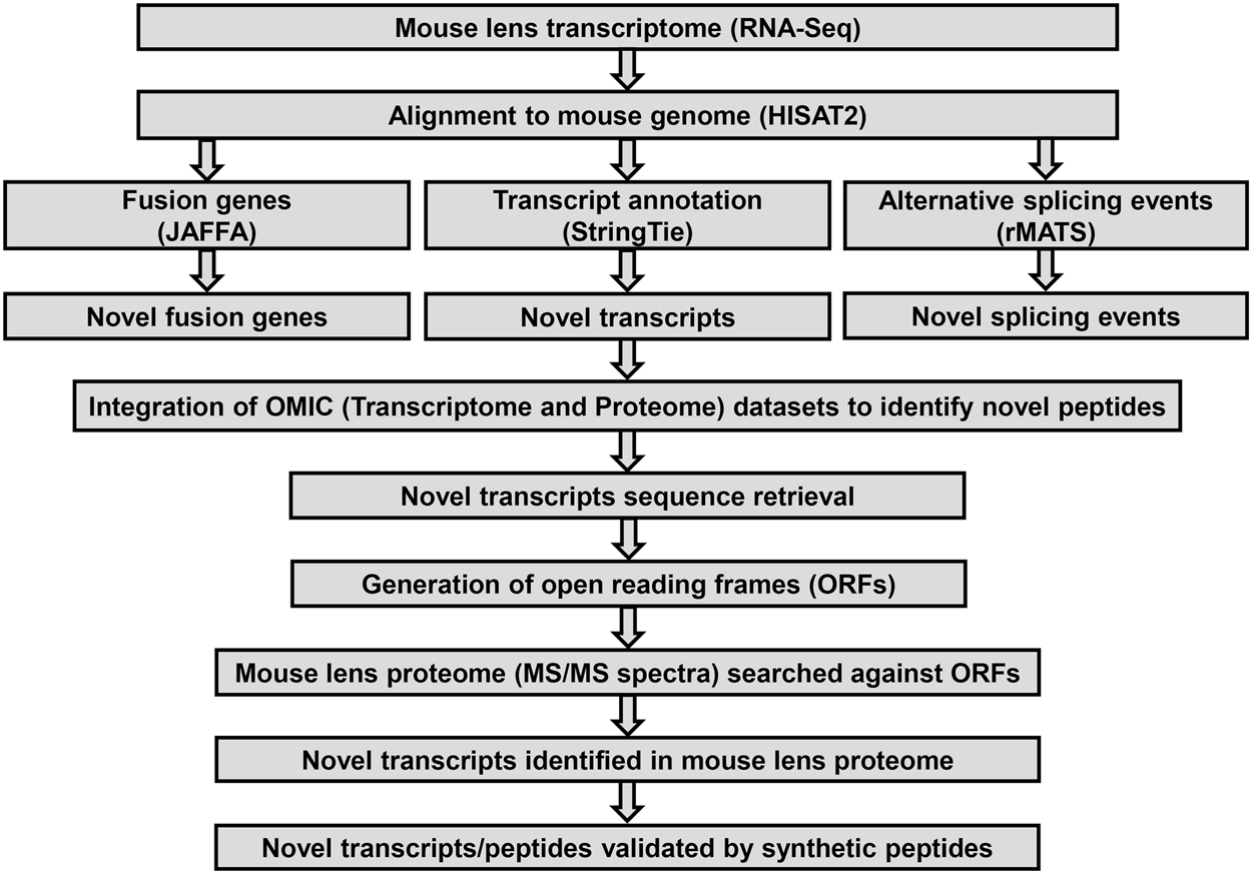
Illustration of the workflow to identify novel transcripts and peptides in developing mouse lens. Briefly, HISAT2, a splice aligner tool was used for the alignment of mouse lens RNA-Seq reads (FASTQ) to the mouse genome, followed by the transcripts annotation and expression quantification using the StringTie algorithm. In parallel, the RNA-Seq aligned data was further processed using JAFFA, and rMATS algorithms to detect fusion genes, and alternative splicing events, respectively, expressing in mouse lens. The novel transcripts (≥1.0TPM) were analyzed using a proteogenomics approach to identify novel peptides. The novel transcripts were translated into potential open reading frames (ORFs) to generate a reference database. The mouse lens proteome data (MS/MS spectra) was searched against this reference database to identify novel peptides. Finally, the novel peptides were validated through matching MS/MS spectra of synthetic peptides.

Here, we extend our analysis of the RNA-Seq data using multiple bioinformatics tools to identify novel transcripts, fusion genes, and alternative splicing (AS) in developing mouse lens (Fig. 1). Additionally, we integrate our OMIC (Transcriptome and Proteome) datasets to identify novel peptides in mouse lens and subsequently validated them through matching MS/MS spectra of synthetic peptides (Fig. 1).

First, the raw reads were mapped to the *Mus musculus* genome resulting in >93% alignment to the genome. Next, the aligned reads were examined for PCR duplicates identifying ~19% of total reads as PCR duplicates that were removed. The remaining mapped reads were processed using StringTie to convert the RNA-Seq alignments into potential transcripts and the expression of each transcript was measured and normalized using transcripts per million (TPM) algorithm. Our analysis identified both annotated and novel transcripts in mouse lens transcriptome. We divided the novel transcripts further into two categories: first, transcripts that map entirely to the unannotated regions of the mouse genome and second, transcripts that partially align to both annotated and unannotated regions of the mouse genome.

We identified a total of 21,265 annotated transcripts expressed in at least one of the six developmental time points (Table 1 & Supplementary Table 1). Of these, we identified 9,707 novel transcripts present in at least one of the six developmental stages mapping entirely to unannotated regions of the mouse genome (Table 1 & Supplementary Table 2). Additionally, we identified 14,113 transcripts aligned to both the annotated and the unannotated regions of the mouse genome (Table 1 & Supplementary Table 3) termed hereafter as semi-novel transcripts.

**Table 1:** Transcripts identified in developing mouse lens.

Developmental Stage	Transcripts expression (≥1.0 TPM)			
	Annotated	Novel	Semi-novel	Fusion Genes
E15	18,087	6,645	11,025	195
E18	17,500	7,061	10,335	161
P0	17,257	7,569	11,194	275
P3	17,257	7,513	11,199	218
P6	16,656	7,293	10,831	280
P9	16,674	7,084	10,605	223
Total	21,265	9,707	14,113	325

Note: E15 and E18 are embryonic days 15, and 18, and P0, P3, P6, and P9 are postnatal days 0, 3, 6, and 9, respectively; TPM: transcripts per million.

We further investigated our mRNA sequencing data to identify fusion genes expressed in the mouse lens transcriptome. The analysis identified 325 novel fusion genes including 195, 161, 275, 218, 280, and 223 fusion genes in the mouse lens at E15, E18, P0, P3, P6, and P9, respectively (Table 1 & Supplementary Table 4). Gene ontologies (GO) based functional and mammalian phenotype enrichment analysis of novel fusion genes revealed (q-value ≤ 0.01) unique molecular function, biological process, cellular component and mammalian phenotypes (Supplementary Tables 5–6).

Next, we examined our RNA-Seq dataset using the rMATS pipeline (≤0.01 FDR) to identify the novel AS events across the six developmental time points in mouse lens. In total, we identified five AS events including exon skipping (ES), alternative 3′ splice site (A3SS), alternative 5′ splice site (A5SS), mutually exclusive exons (MXE), and intron retention (IR) in developing mouse lens (Table 2). The analysis identified 6,990 novel ES splicing events (≤0.01 FDR) across the six developmental time points in mouse lens (Supplementary Table 7). Of these 2,023 events present in at least one developmental time point, and 4,967 ES events in ≥2-time points (Supplementary Table 7).

**Table 2:** Alternative splicing events identified in developing mouse lens.

Developmental Stage	Types of AS Events	Total AS Events	Significant Novel AS Events*
E15 vs. E18	ES	22160	416
	MXE	3069	93
	A5SS	9753	162
	A3SS	13755	306
	IR	575	23
E15 vs. P0	ES	26560	521
	MXE	4379	124
	A5SS	10743	97
	A3SS	14818	93
	IR	646	11
E15 vs. P3	ES	23261	336
	MXE	3881	107
	A5SS	10247	106
	A3SS	14215	107
	IR	605	8
E15 vs. P6	ES	22898	459
	MXE	3827	129
	A5SS	10119	110
	A3SS	14093	119
	IR	617	8
E15 vs. P9	ES	21982	444
	MXE	3872	121
	A5SS	9859	97
	A3SS	13623	94
	IR	559	13
E18 vs. P0	ES	23579	1107
	MXE	3470	194
	A5SS	9295	259
	A3SS	12849	309
	IR	557	26
E18 vs. P3	ES	20212	810
	MXE	3321	219
	A5SS	8888	233
	A3SS	12271	287
	IR	515	19
E18 vs. P6	ES	19789	931
	MXE	3109	198
	A5SS	80692	238
	A3SS	12123	300
	IR	503	21
E18 vs. P9	ES	18776	921
	MXE	2938	188
	A5SS	8384	241
	A3SS	11645	290
	IR	452	15
P0 vs. P3	ES	24298	281
	MXE	4296	131
	A5SS	9669	82
	A3SS	13227	108
	IR	586	5
P0 vs. P6	ES	23843	194
	MXE	4265	33
	A5SS	9581	46
	A3SS	13119	88
	IR	583	6
P0 vs. P9	ES	22985	214
	MXE	4137	49
	A5SS	9293	67
	A3SS	12681	114
	IR	533	9
P3 vs. P6	ES	20632	149
	MXE	4121	108
	A5SS	9142	62
	A3SS	12526	89
	IR	542	3
P3 vs. P9	ES	19721	143
	MXE	3816	60
	A5SS	8832	54
	A3SS	12003	78
	IR	499	4
P6 vs. P9	ES	19208	64
	MXE	4083	17
	A5SS	8672	46
	A3SS	11934	65
	IR	492	2

Note: AS: alternative splicing; ES: exon skipping; MXE: mutually exclusive exon; A5SS: alternative 5′ splice site; A3SS: alternative 3′ splice site; and IR: intron retention. *Significant events based on false discovery rate (FDR) <0.01. E15 and E18 are embryonic days 15, and 18, and P0, P3, P6, and P9 are postnatal days 0, 3, 6, and 9, respectively.

We identified 2,447 novel A3SS splicing events (≤0.01 FDR) including 809 events detected in a single developmental time point and 1,638 events in ≥2-time points (Supplementary Table 8). Likewise, we identified 1,900 novel A5SS splicing events including 719 events detected in a single developmental time point and 1,181 events in ≥2-time points (Supplementary Table 9). Furthermore, we identified 1,771 novel MXE splicing events (≤0.01 FDR) including 387 events detected in a single developmental time point and 1,384 events in ≥2-time points (Supplementary Table 10). Lastly, our analysis identified 173 IR splicing events (≤0.01 FDR) in mouse lens (Supplementary Table 11).

Our RNA-Seq datasets are critical in identifying novel transcripts; however, the biological significance of these events is incomplete without knowing the corresponding changes at the protein level. We recently investigated the proteome profile of developing mouse lens through mass spectrometry-based protein sequencing^24^. We integrated our OMIC datasets to identify novel peptides in mouse lens. As mentioned above, we identified a total of 9,707 novel transcripts that were translated into three open reading frames (ORFs) to identify all theoretical peptides translated by the novel transcripts. This theoretical peptide dataset was interrogated against the mouse lens proteome to identify peptides originating from a sequence of the novel transcripts (9,707 novel transcripts identified in the mouse lens transcriptome). The analysis identified 55 peptides in the mouse lens proteome based on TMT spectra. All of the 55 candidate peptides were screened against the mouse non-redundant (nr) protein database (NCBI) and peptides with ≥2 amino acids mismatches and an XCorr score ≥2.5 were considered novel. This criterion identified a total of 20 novel peptides that were retained for further analysis.

All 20 novel peptides along with three control peptides were synthesized commercially and the respective spectra of these synthetic peptides were generated using the Orbitrap Fusion Lumos Tribrid Mass Spectrometer. The MS/MS fragmentation patterns of synthetic peptides were manually compared with MS/MS spectra generated from the proteomic analysis of mouse lens. The control peptides revealed similar spectra consistent with the MS/MS fragmentation patterns originating from mouse lens proteome (Supplementary Figs 1–3). The MS/MS fragmentation patterns of all 20 synthetic peptides (representing 20 novel peptides) exhibited spectrum consistent the MS/MS fragmentation patterns originating from mouse lens proteome dataset (Table 3, Fig. 2, Supplementary Figs 4–20, and Supplementary Table 12).

**Table 3:** Novel peptides identified in mouse lens through integration of OMIC (Transcriptome and Proteome) datasets^22,24^.

No.	Novel Peptide	Amino Acid Length	Number of PSMs	Novel Transcript ID	Genomic Coordinates (mm10)
1	AAESDLSTARPAPPEPR	17	1	MSTRG.4120.3	Chr11: 117620218–117620288
2	AFAHAEPR	8	2	MSTRG.6156.3	Chr13: 120244503–120244596
3	ATEDCFQER	9	2	MSTRG.5109.18	Chr12: 113023646–113023713
4	AVGVDCSAPEPR	12	2	MSTRG.3713.5	Chr11: 97361423–97363714
5	DLGGVESASPSAAR	14	1	MSTRG.19717.4	Chr7: 137903048–137903154
6	DREIWLNR	8	4	MSTRG.12904.4	Chr2: 177493993–177494080
7	GLQRPDGGDHR	11	2	MSTRG.398.2	Chr1: 65973222–65973464
8	LCGACGTASGTK	12	3	MSTRG.16432.2	Chr5: 112688277–112688395
9	LRHLNNVNILK	11	2	MSTRG.11709.1	Chr2: 38729037–38735032
10	NYFYTGAEIK	10	1	MSTRG.19785.1	Chr7: 146523115–146545389
11	QGISSISTFK	10	3	MSTRG.18410.1	Chr7: 19857400–19857609
12	SAQALVK	7	2	MSTRG.6483.20	Chr14: 48326083–48326173
13	SINEVIK	7	1	MSTRG.5839.2	Chr13: 74088982–74089093
14	SMGEDTVPK	9	1	MSTRG.6973.3	Chr14: 121639196–121639264
15	WLIEISK	7	3	MSTRG.8658.5	Chr17: 3516990–3517538
16	FLTVSTSPGFPGT	13	1	MSTRG.16436.1	Chr5: 112684967–112698364
17	AAGDAEPEDQAQPQPQPEPEPR	22	4	MSTRG.5528.4	Chr13: 46517220–46517274
18	LGHVGGADGANPSSAGSPQDGR	22	1	MSTRG.3412.1	Chr11: 77839387–77839530
19	SPGSEPQTQEAQEAGSDPQAARPQR	25	2	MSTRG.18532.2	Chr7: 28289684–28289782
20	ERPTPDVGDGQGPQLSESSSSPFSIPPDK	29	11	MSTRG.12232.3	Chr2: 120330209–120330655

All 20 peptides were validated through matching MS/MS spectra of synthetic peptides. Note: PSMs: Peptide Spectrum Matches.

**Figure 2:**
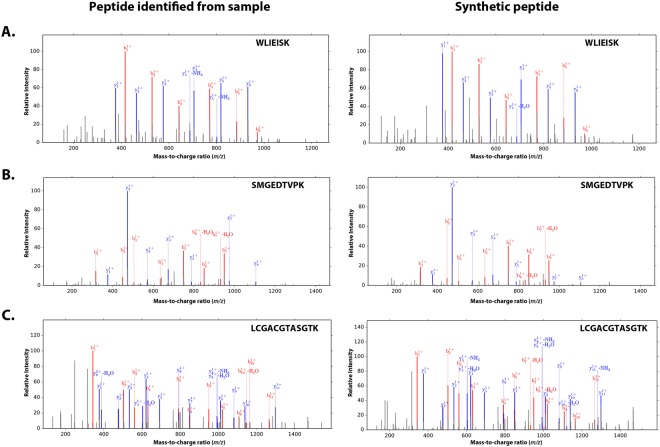
Validation through matching MS/MS spectra of synthetic peptides. (**A**) MS/MS spectra of a novel peptide (WLIEISK) shown with a similar fragmentation pattern observed from the corresponding synthetic peptide. (**B**) MS/MS spectra of a novel peptide (SMGEDTVPK) shown with a similar fragmentation pattern observed from the corresponding synthetic peptide. (**C**) MS/MS spectra of a novel peptide (LCGACGTASGTK) shown with a similar fragmentation pattern observed from the corresponding synthetic peptide. Note: the term “peptide identified from sample” refers to the MS/MS spectra identified in mouse lens proteome^24^, and the synthetic peptide refers to MS/MS spectra of the peptide synthesized by JPT Peptide Technologies (Berlin, Germany).

## Discussion

We previously investigated mouse lens transcriptome at two embryonic (E15 and E18) and four postnatal (P0, P3, P6, and P9) time points using next-generation RNA sequencing, which identified a total of 14,465 genes along with 12 different classes of non-coding RNAs (ncRNAs) in mouse lens^22,23^. More recently, we completed a comprehensive proteome of mouse lens at the same six developmental time points identifying 5,404 proteins^24^. A brief overview of these published datasets is provided in Table 4. In here, we extend our analyses to identify novel transcripts and novel peptides in developing mouse lens.

**Table 4:** Data retrieved from previously published OMIC (Transcriptome and Proteome) datasets^22,24^.

A. Mouse Lens Transcriptome (RNA-Seq)				B. Mouse Lens Proteome (TMT)			
Stage	Replicates	Genes	Total Genes	Stage	TMT	Proteins	Total Proteins
E15	E15A E15B	13,274	14,465	E15	Set 1	4,630	5,404
				E18			
				P0			
E18	E18A E18B	13,900		P3			
				P6			
				P9			
P0	P0A P0B	12,560		E15	Set 2	4,426	
				E18			
				P0			
P3	P3A P3B	12,940		P3			
				P6			
				P9			
P6	P6A P6B	12,130		E15	Set 3	3,747	
				E18			
				P0			
P9	P9A P9B	12,229		P3			
				P6			
				P9			

Note: E15 and E18 are embryonic days 15, and 18, and P0, P3, P6, and P9 are postnatal days 0, 3, 6, and 9, respectively.

Our analysis revealed 9,707 novel transcripts identified in six developmental time points (Table 1, and Supplementary Table 2). Of these, ~25% are multi-exonic and ~40% are expressed in all six developmental time points (Supplementary Table 2). In addition to novel transcripts, we identified a total of 14,113 semi-novel transcripts in lens transcriptome (Table 1, and Supplementary Table 3). In contrast to the novel transcripts where a majority (~75%) of the transcript are single exon, most of the semi-novel transcripts (>85%) revealed a multi-exonic structure i.e. ≥2.0 exons (Supplementary Tables 2 and 3).

We identified 325 novel fusion genes in developing mouse lens (Table 1). Among these, we identified multiple fusion transcripts for *CryαA*, *CryαB*, *CryβA1*, *CryβA2*, *CryβA4*, *CryβB1*, *CryβB2*, *CryβB3*, *CryƔA*, *CryƔC*, *CryƔD*, *CryƔE*, and *CryƔS* (Supplementary Table 4). Additionally, we identified fusion transcripts for *Bfsp1*, *Bfsp2*, *Tdrd7*, *Mip*, *Lim2*, *Pax6*, and *Dnase2β* in mouse lens (Supplementary Table 4). Multiple studies have reported fusion genes in normal human and mouse tissues and more importantly, in tumorigenesis^25–28^. We did not find any reports in literature, and therefore this would be the first report describing fusion genes in the ocular lens.

Multiple transcriptome-based studies have revealed extensive AS in normal human and mouse ocular tissues^29–35^. We identified 13,281 novel AS events in developing mouse lens with a predominant contribution from ES, A3SS, A5SS, and MXE while a small number of IR splicing events identified in developing mouse lens (Table 2). The ES events contribute to the diversity of the transcriptome and consistent with this notion, we identified a total of 6,990 ES events in developing mouse lens. Interestingly, most of the ES were identified at embryonic time points (Table 2).

Recently, Srivastava and colleagues reported the identification of 1,241 AS events in developing mouse lens^21^, examining RNA-Seq datasets recently published by our group^22^. We identified a total of 13,281 AS events in developing mouse lens analyzing the same RNA-Seq datasets. The difference in the numbers of AS events published by Srivastava and colleagues and identified in the current study may be attributed to different cut-off values. Srivastava and colleagues used PSI (Percent Spliced Index) cut-off value for significant (<1% FDR) AS events^21^, whereas we used a cut-off value of ≤0.01 FDR for the identification of AS events in developing mouse lens.

Finally, we adopted a proteogenomics approach to identify expression of novel transcripts in mouse lens proteome and subsequently validated these novel peptides through MS/MS spectra of corresponding synthetic peptides. In silico analyses identified a total of 55 novel peptides in mouse lens protome. Of these, only 20 peptides passed a more stringent criterium i.e. ≥2 amino acids mismatches and an XCorr score ≥2.5. These 20 novel peptides were validated through MS/MS spectra of corresponding synthetic peptides. We are currently investigating the identity of the proteins harboring these novel peptides and examining their biological significance in lens morphogenesis.

In conclusion, we represent a comprehensive developing mouse lens profile through the identification of novel transcripts, novel fuson genes, and novel AS events. Additionally, we integrate our OMIC datasets to identify novel peptides in developing mouse lens.

## Materials and Methods

### mRNA sequencing data

The Illumina paired-end mRNA sequencing data (GEO series accession number GSE69221) was used for the downstream bioinformatics analysis. The paired-end raw reads were processed to remove the adapter sequences using SeqPrep (https://github.com/jstjohn/SeqPrep). The quality of the pre-processed reads was evaluated with FastQC (www.bioinformatics.babraham.ac.uk/projects/fastqc) and low-quality reads were removed prior to the downstream analysis.

### Mapping and transcript annotation of mRNA sequencing data

HISAT2 (Hierarchical Indexing for Spliced Alignment of Transcripts), a spliced alignment tool (Ver. 2.1.0-beta) was used to map pre-processed reads to the mouse genome (NCBI37/mm9) with default parameter settings^36^. The BAM output files were generated for each sample and PCR duplicates were marked and removed from BAM files using Picard software (Ver. 2.8.3; https://github.com/broadinstitute/picard). Subsequently, StringTie algorithm (Ver. 1.3.3b) was used with default parameter settings to assemble RNA-Seq alignments into annotated and novel transcripts and estimate their respective expression level^37^. The expression of these transcripts was normalized using transcripts per million (TPM) algorithm and the number of known, and novel transcripts were estimated from the output GTF file generated by StringTie with expression threshold (≥1.0 TPM).

### Identification of AS events

AS events were identified through rMATS software (Ver. 3.2.5)^38^. The mRNA-Seq alignment files (bam files) generated by the HISAT2 was used as an input for the rMATS analysis. The Mus musculus RefSeq gene annotations (GRcm38/mm10) was used as a reference with default parameter settings. Finally, the rMATS was used to calculate *p*-value and false discovery rate (FDR) for AS events among different developmental time points.

### Identification of fusion genes

JAFFA (Ver. 1.08), a multi-step pipeline was used in a hybrid mode to detect fusion genes in mRNA sequencing data^39^. The Mus musculus reference genome (GRcm38/mm10), transcripts annotations and sequences from mouse GENCODE Ver. M15 (GRCm38) were used as a reference for fusion gene identification using default JAFFA parameter settings.

### Gene ontologies functional enrichment analysis

A functional annotation analysis of mouse lens genes was investigated using Visual Annotation Display (VLAD; Ver. 1.6.0), a web-based tool from the Mouse Genome Informatics (MGI)^40^. The VLAD tool performs the statistical analysis to test the enrichment of gene ontology (GO) terms based on their annotations to gene function and mammalian phenotype^40^. A complete set of mouse genes was used as a reference annotation dataset and ontological terms annotated with the evidence code ND (no biological data) were excluded from the enrichment analysis. The statistically significant enriched terms were sorted based on their corrected *p*-value (≤0.01) calculated using multiple testing and positive false discovery rate for each term.

### Proteogenomic analysis of novel transcripts in lens proteome

The mRNA sequencing data was used to extract Fasta sequences of novel transcripts using bedtools getfasta tool (Ver. 2.25.0; http://bedtools.readthedocs.io/en/latest/content/tools/getfasta.html) with the transcript coordinates output by StringTie. The novel transcripts were translated into three open reading frames (ORFs) using in-house generated Python script to generate a dataset of all potential proteins resulting from the translation of the novel transcripts. The theoretical protein dataset was screened to remove shorter proteins (<6 amino acids long) and the resulting dataset was used as a reference database.

The MS/MS spectra from mouse lens proteome were interrogated against the reference database (generated above, using the Python script) using the SEQUEST search algorithm through the Proteome Discoverer Suite (Ver. 2.1; Thermo Scientific, Bremen, Germany). The parameters for the Proteome Discoverer included, trypsin as a proteolytic enzyme with a maximum of two missed cleavages, acetylation of protein N-termini and oxidation of methionine as variable modifications, carbamidomethylation of cysteine as fixed modification, a minimum peptide length of 6 amino acids, and the mass tolerances of 10 ppm and 0.02 Da for precursor and fragment ions, respectively. The matched spectra or candidate peptides were filtered using the Percolator algorithm within the Proteome Discoverer suite using the false discovery rate (FDR < 0.01) at protein and peptide levels.

Finally, all peptides identified above (by interrogating the MS/MS spectra from mouse lens proteome against the reference database) were screened against the mouse nr protein database (NCBI) to identify novel peptides not present in the mouse nr protein database. Peptides with ≥2 amino acids mismatches (mouse nr protein database) and XCorr score ≥2.5 were considered novel and retained for further analysis.

### Validation of novel peptides through LC-MS/MS analysis of synthetic peptides

All synthetic peptides were purchased from JPT Peptide Technologies (Berlin, Germany). The synthetic peptides were pooled and labeled with 1-plex TMT reagents according to the manufacturer’s instructions (Thermo Fisher Scientific). The labeling reaction was performed for one hour at room temperature followed by quenching of the labeling reaction with 100 mM Tris-HCl (pH 8.0). The labeled peptides were desalted with C_18_ Sep-Pak (Waters Corporation, Milford, MA), dried and resuspended in 0.1% formic acid. Peptides were subjected to Orbitrap Fusion Lumos Tribrid Mass Spectrometer coupled with the Easy-nLC 1200 nano-flow liquid chromatography system (Thermo Fisher Scientific) with similar parameters used for the mouse lens proteome profiling^24^.

## Electronic supplementary material

Supplementary Information

Supplementary Table 1

Supplementary Table 2

Supplementary Table 3

Supplementary Table 4

Supplementary Table 5

Supplementary Table 6

Supplementary Table 7

Supplementary Table 8

Supplementary Table 9

Supplementary Table 10

Supplementary Table 11

Supplementary Table 12
