# Supplementary material for: Identification of novel transcripts and peptides in developing murine lens
Source: Sci Rep. 2018 Jul 24;8:11162. doi: 10.1038/s41598-018-28727-w (PMC6057992; doi:10.1038/s41598-018-28727-w)
Supplement: Supplementary file 1 — Supplementary Information [file 41598_2018_28727_MOESM1_ESM.docx]

**Identification of novel transcripts and peptides in developing murine lens**

Shahid Y. Khan,^1^ Muhammad Ali,^1^ Firoz Kabir,^1^ Ruiqiang Chen,^2^ Chan Hyun Na,^2^ Mei-Chong Wendy Lee,^3^ Nader Pourmand,^3^ Sean F. Hackett,^1^ S. Amer Riazuddin^1^

^1^The Wilmer Eye Institute, Johns Hopkins University School of Medicine, Baltimore, MD 21287; ^2^Department of Biological Chemistry, Johns Hopkins University School of Medicine, Baltimore, MD 21205; ^3^Department of Biomolecular Engineering, University of California, Santa Cruz, CA 94305

**Correspondence to:**

S. Amer Riazuddin, Ph.D.

The Wilmer Eye Institute

Johns Hopkins University School of Medicine

600 N. Wolfe Street; Maumenee 809

Baltimore, MD 21287 USA

Email: riazuddin@jhmi.edu

**Funding support:** The work was supported by National Eye Institute Grant R01EY022714 (SAR).

**Content**

**­________________________________________________________________________**

Contents .…..………………………………………………………………….…………..………...2

Supplementary Figure 1…………………...………………………………………….……………3

Supplementary Figure 2 …….…………………………………………….………….……………4

Supplementary Figure 3 …….…………………………………………….………….……………5

Supplementary Figure 4……………………………………………………………………………6

Supplementary Figure 5……………………………………………………………………………7

Supplementary Figure 6……………………………………………………………………………8

Supplementary Figure 7……………………………………………………………………………9

Supplementary Figure 8……………………………………………………………………………10

Supplementary Figure 9……………………………………………………………………………11

Supplementary Figure 10 ………………………………………….……………………...………12

Supplementary Figure 11 ………………………………………….……………………...………13

Supplementary Figure 12 ………………………………………….……………………...………14

Supplementary Figure 13 ………………………………………….……………………...………15

Supplementary Figure 14 ………………………………………….……………………...………16

Supplementary Figure 15 ………………………………………….……………………...………17

Supplementary Figure 16 ………………………………………….……………………...………18

Supplementary Figure 17 ………………………………………….……………………...………19

Supplementary Figure 18 ………………………………………….……………………...………20

Supplementary Figure 19 ………………………………………….……………………...………21

Supplementary Figure 20 ………………………………………….……………………...………22

Legend for Supplementary Tables 1 - 12 ……………...……...……….…………………….... 23 - 25

**
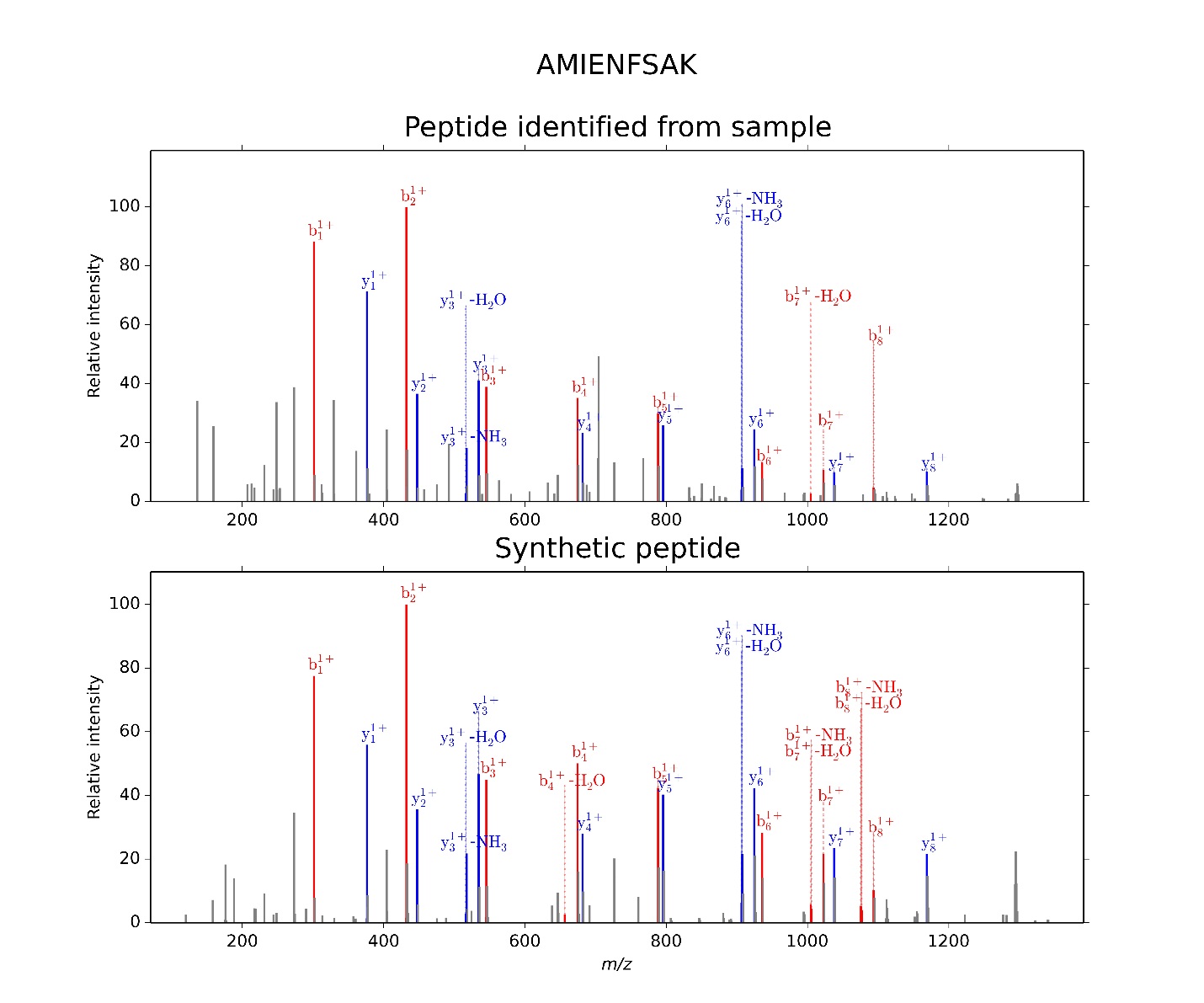
**

**Supplementary Figure 1.** The validation of MS/MS spectra of the control peptide (AMIENFSAK) identified in mouse lens proteome shown along with a similar fragmentation pattern observed from the corresponding synthetic peptide. **Note:** the term “peptide identified from sample” refers to the MS/MS spectra identified in mouse lens proteome (Khan et al. *Invest Ophthalmol Vis Sci*. 2018;59:100-107), and the synthetic peptide refers to MS/MS spectra of the peptide synthesized by JPT Peptide Technologies (Berlin, Germany).


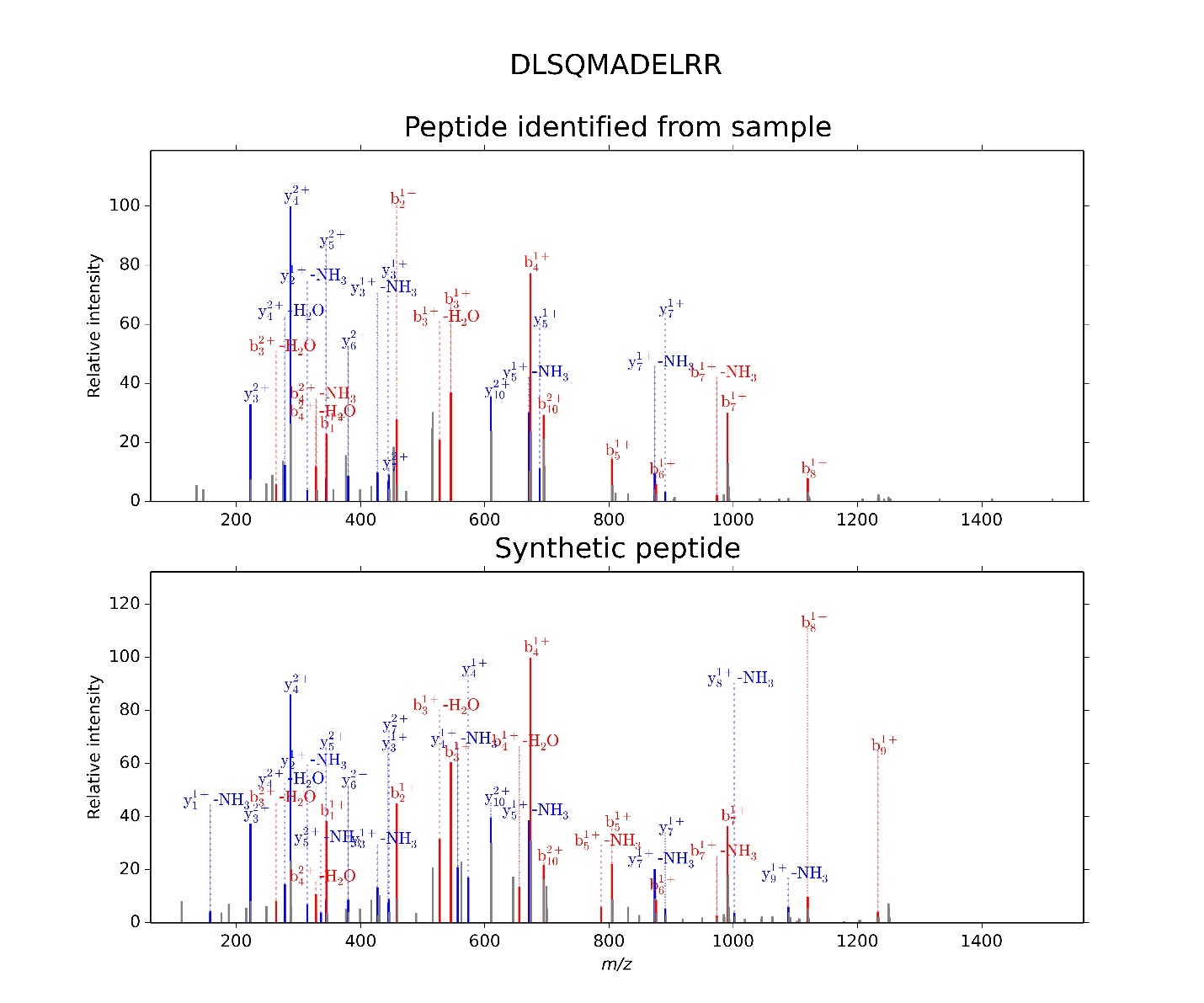


**Supplementary Figure 2.** The validation of MS/MS spectra of the control peptide (DLSQMADELRR) identified in mouse lens proteome shown along with a similar fragmentation pattern observed from the corresponding synthetic peptide. **Note:** The terms “peptide identified from sample” and “synthetic peptide” are described in Supplementary Figure 1.


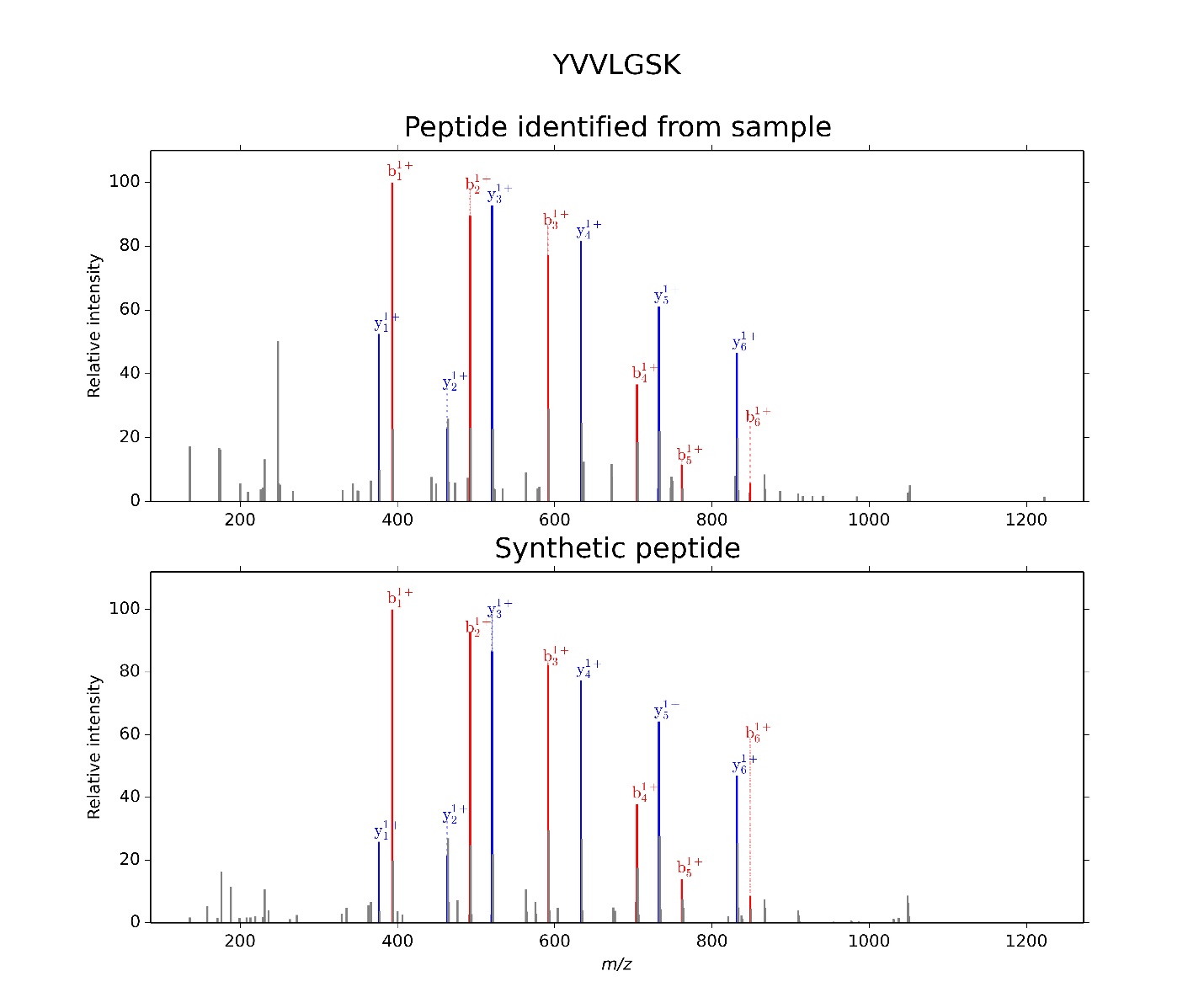


**Supplementary Figure 3.** The validation of MS/MS spectra of the control peptide (YVVLGSK) identified in mouse lens proteome shown along with a similar fragmentation pattern observed from the corresponding synthetic peptide. **Note:** The terms “peptide identified from sample” and “synthetic peptide” are described in Supplementary Figure 1.


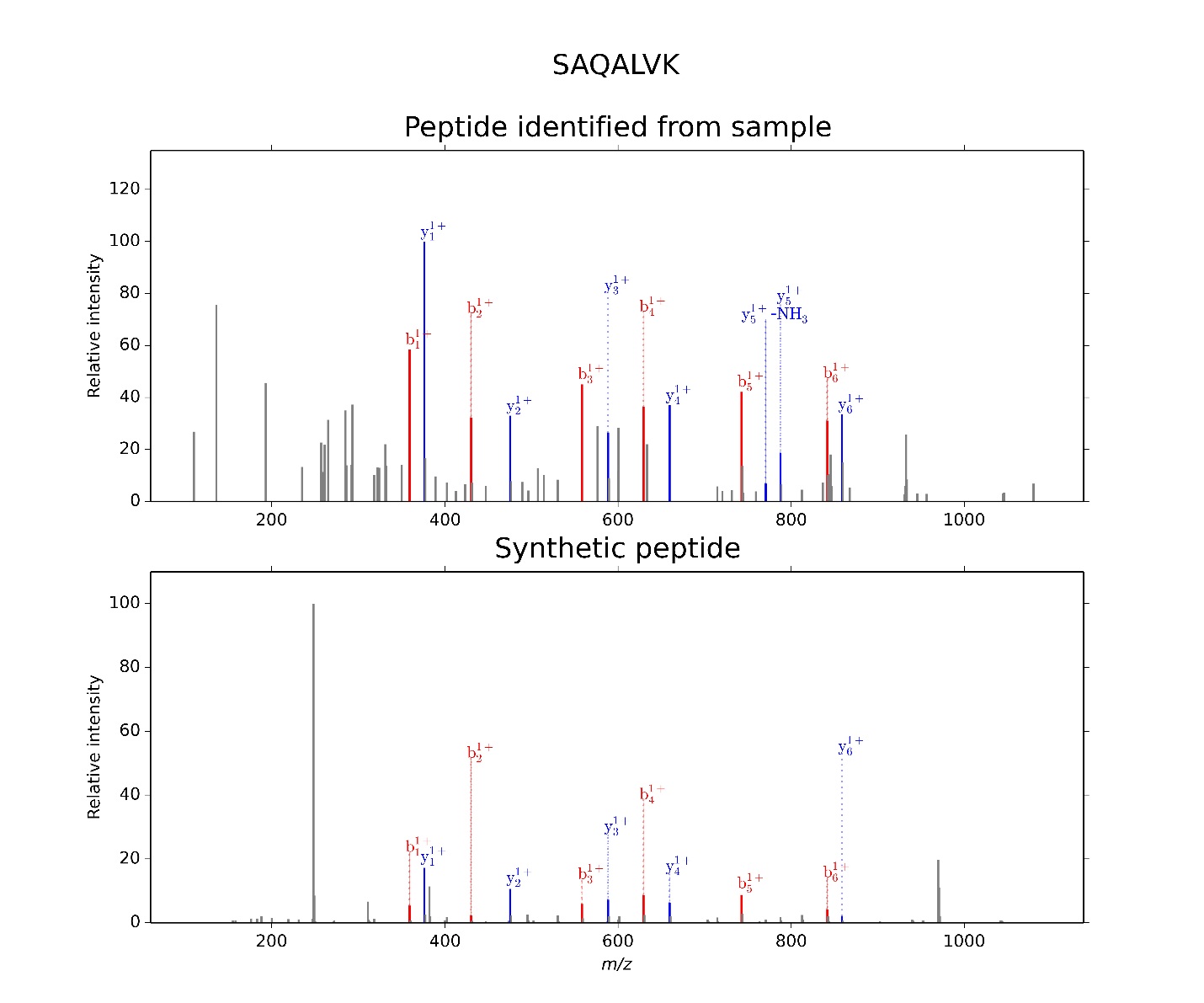


**Supplementary Figure 4.** The validation of MS/MS spectra of the novel peptide (SAQALVK) identified in mouse lens proteome shown along with a similar fragmentation pattern observed from the corresponding synthetic peptide. **Note:** The terms “peptide identified from sample” and “synthetic peptide” are described in Supplementary Figure 1.


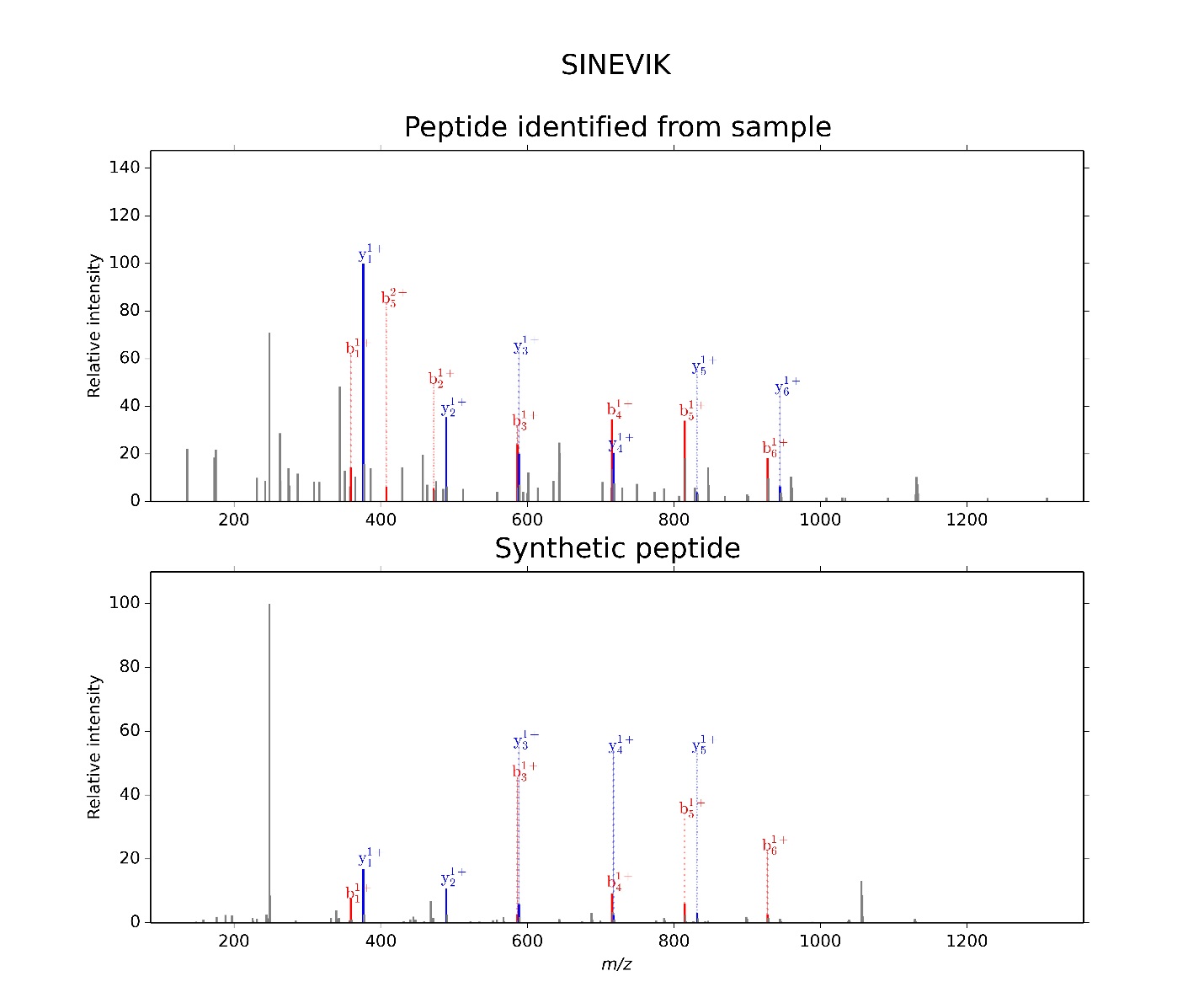


**Supplementary Figure 5.** The validation of MS/MS spectra of the novel peptide (SINEVIK) identified in mouse lens proteome shown along with a similar fragmentation pattern observed from the corresponding synthetic peptide. **Note:** The terms “peptide identified from sample” and “synthetic peptide” are described in Supplementary Figure 1.


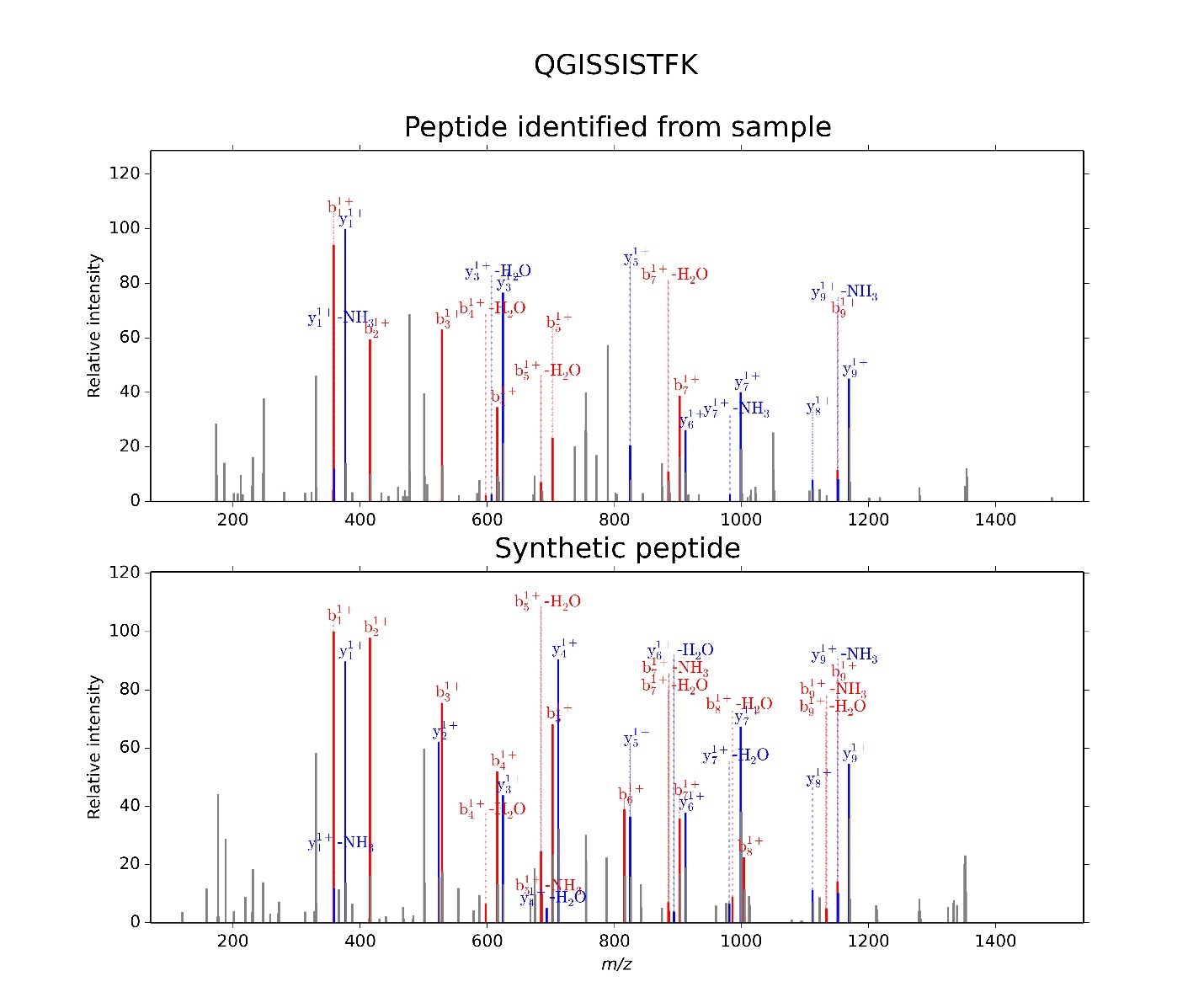


**Supplementary Figure 6.** The validation of MS/MS spectra of the novel peptide (QGISSISTFK) identified in mouse lens proteome shown along with a similar fragmentation pattern observed from the corresponding synthetic peptide. **Note:** The terms “peptide identified from sample” and “synthetic peptide” are described in Supplementary Figure 1.


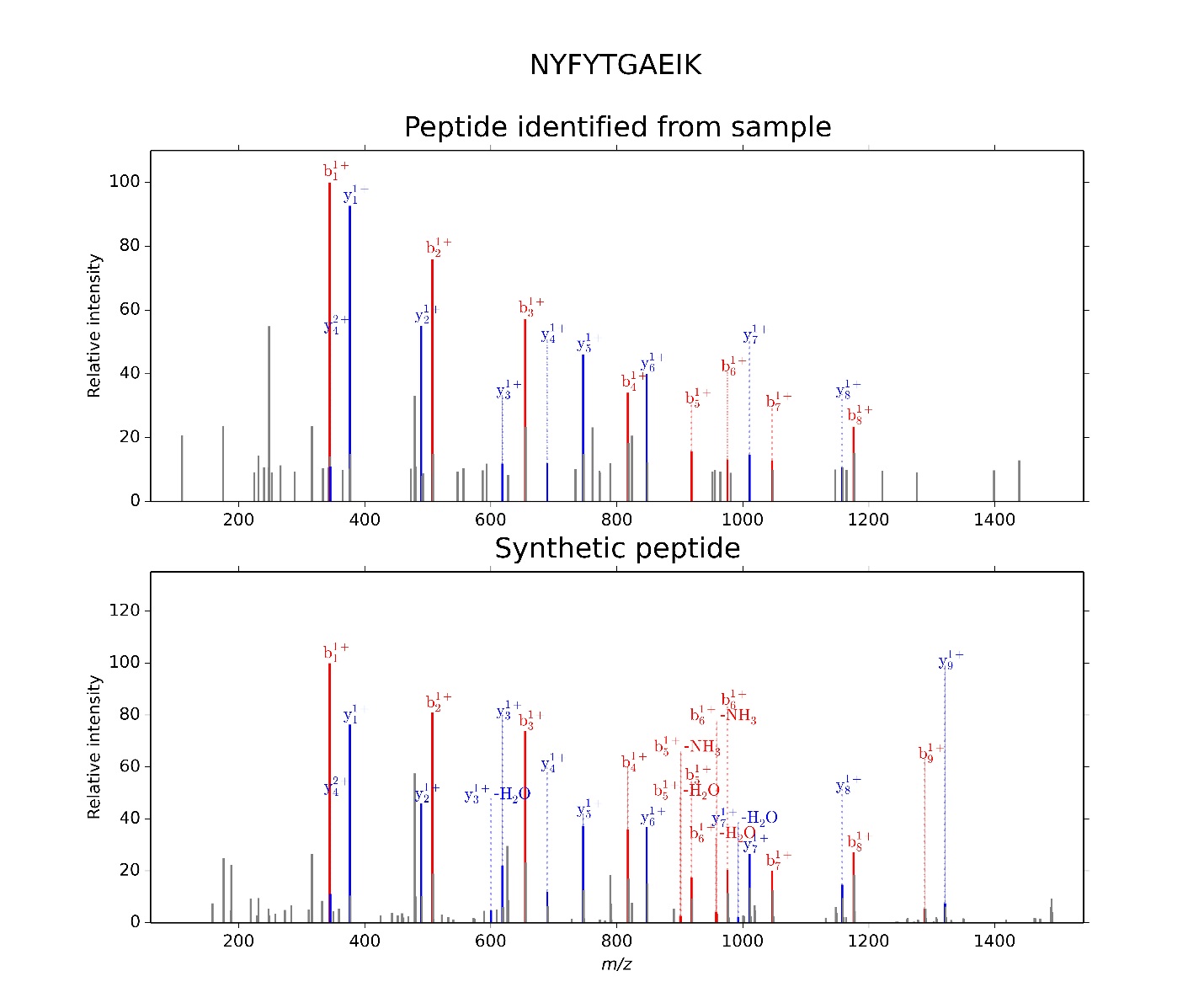


**Supplementary Figure 7.** The validation of MS/MS spectra of the novel peptide (NYFYTGAEIK) identified in mouse lens proteome shown along with a similar fragmentation pattern observed from the corresponding synthetic peptide. **Note:** The terms “peptide identified from sample” and “synthetic peptide” are described in Supplementary Figure 1.


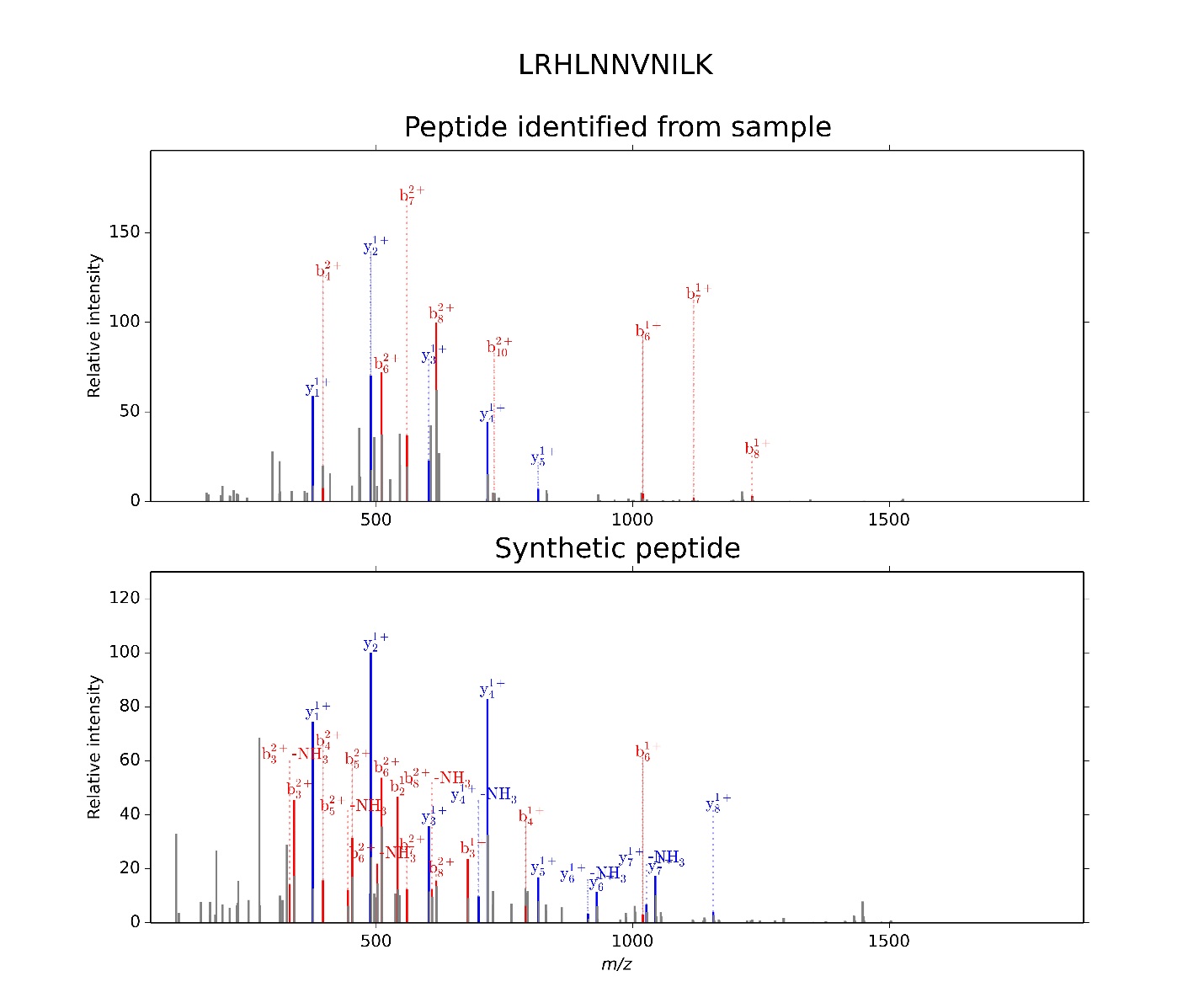


**Supplementary Figure 8.** The validation of MS/MS spectra of the novel peptide (LRHLNNVNILK) identified in mouse lens proteome shown along with a similar fragmentation pattern observed from the corresponding synthetic peptide. **Note:** The terms “peptide identified from sample” and “synthetic peptide” are described in Supplementary Figure 1.


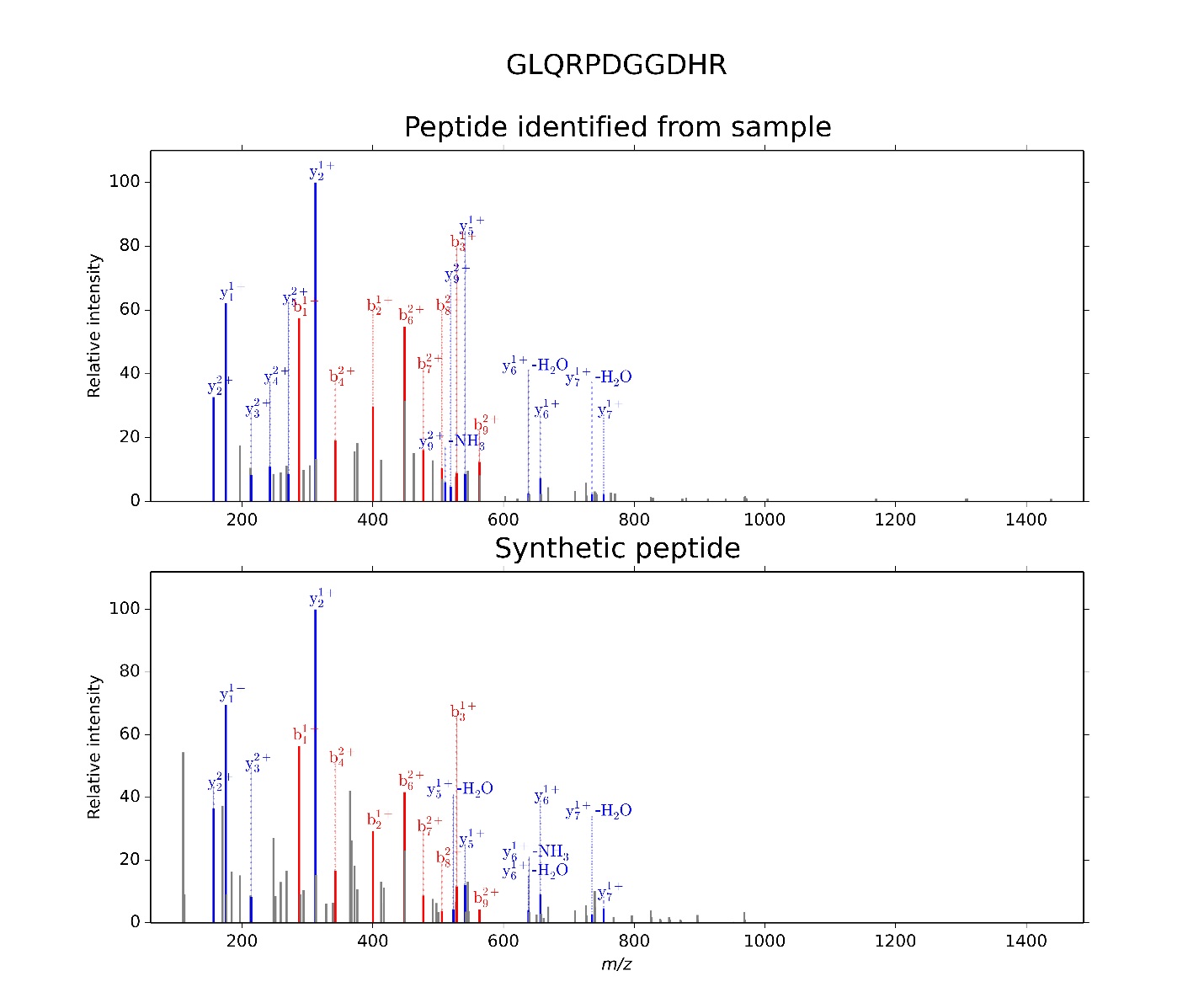


**Supplementary Figure 9.** The validation of MS/MS spectra of the novel peptide (GLQRPDGGDHR) identified in mouse lens proteome shown along with a similar fragmentation pattern observed from the corresponding synthetic peptide. **Note:** The terms “peptide identified from sample” and “synthetic peptide” are described in Supplementary Figure 1.


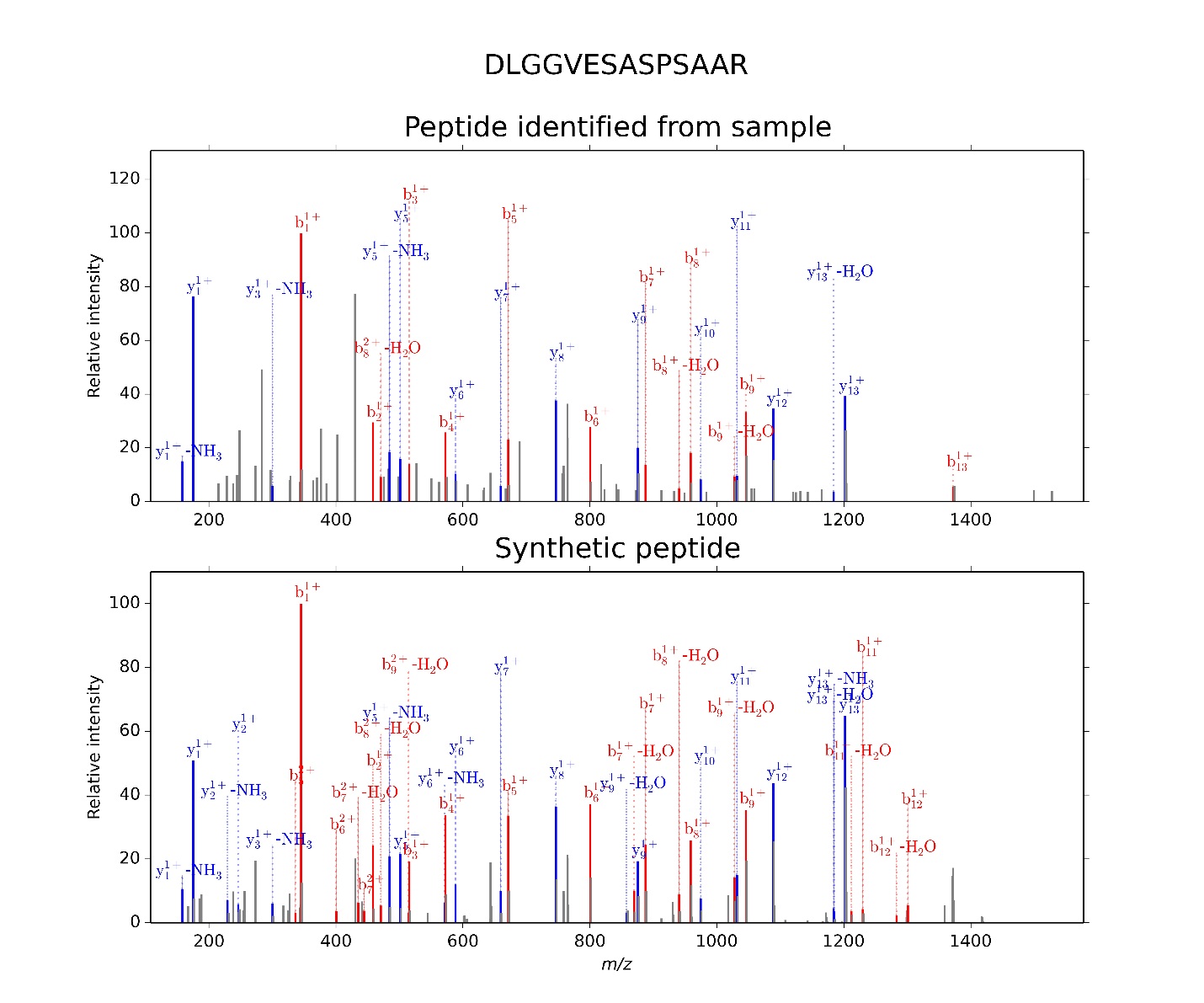


**Supplementary Figure 10.** The validation of MS/MS spectra of the novel peptide (DLGGVESASPSAAR) identified in mouse lens proteome shown along with a similar fragmentation pattern observed from the corresponding synthetic peptide. **Note:** The terms “peptide identified from sample” and “synthetic peptide” are described in Supplementary Figure 1.


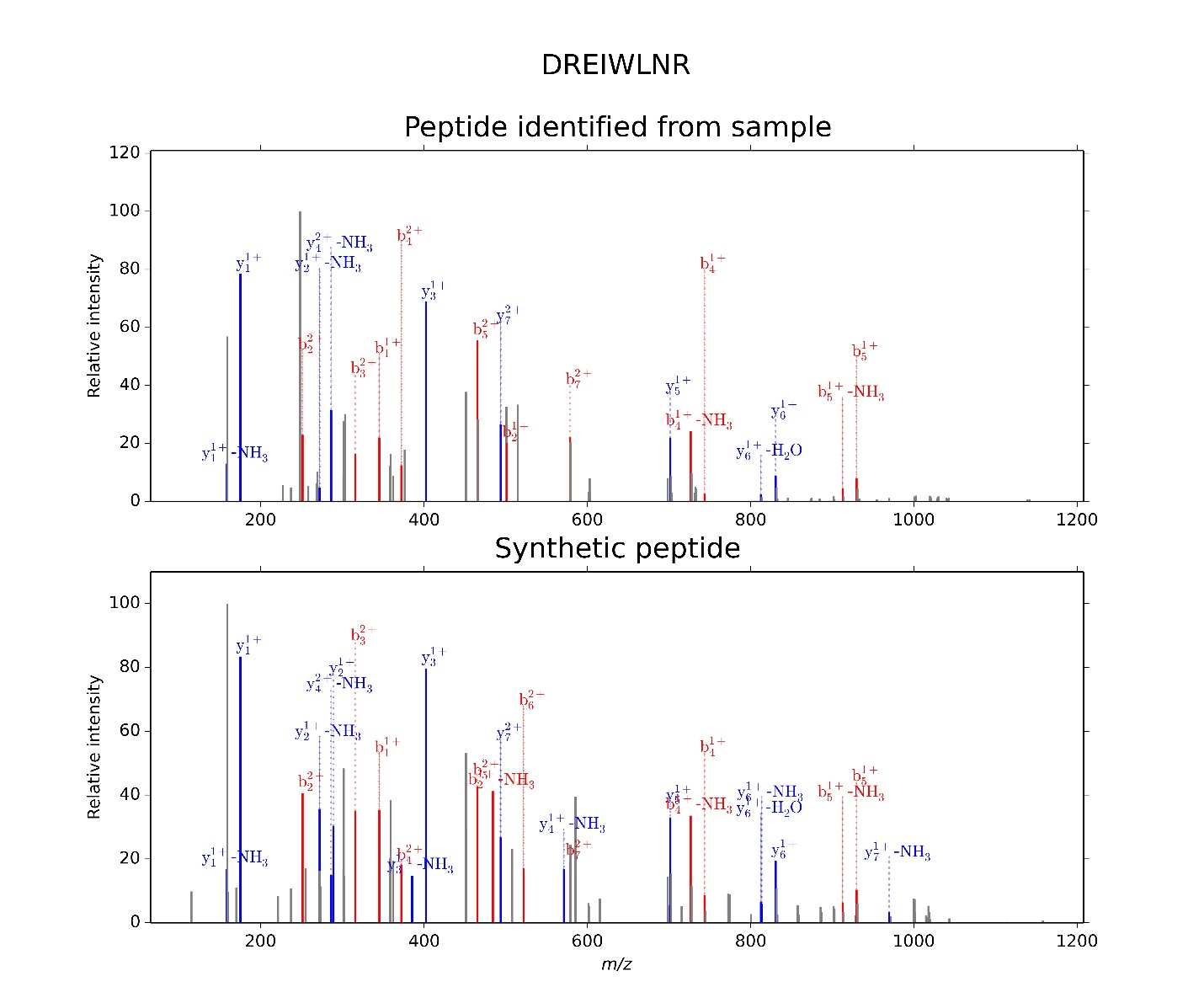


**Supplementary Figure 11.** The validation of MS/MS spectra of the novel peptide (DREIWLNR) identified in mouse lens proteome shown along with a similar fragmentation pattern observed from the corresponding synthetic peptide. **Note:** The terms “peptide identified from sample” and “synthetic peptide” are described in Supplementary Figure 1.


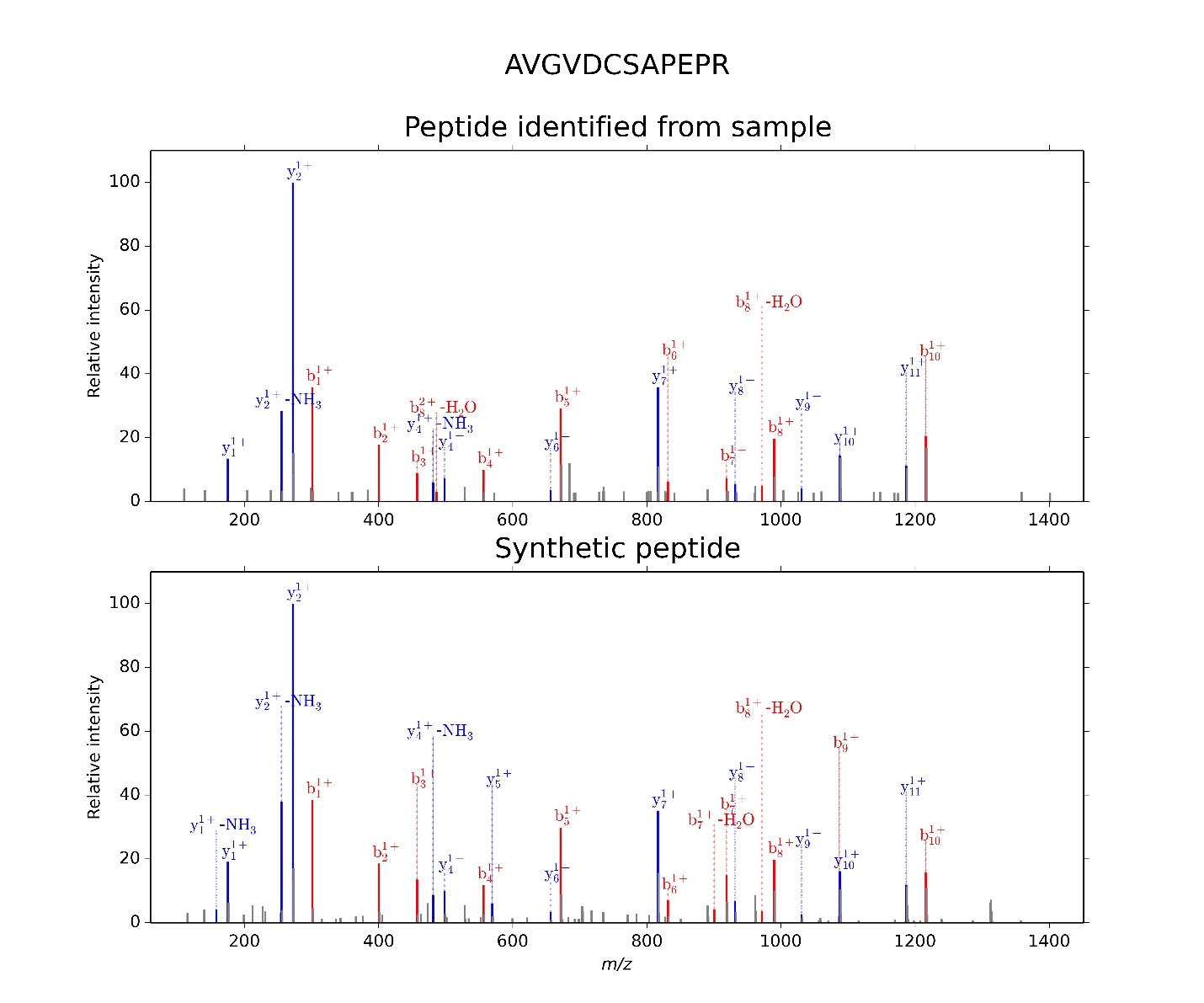


**Supplementary Figure 12.** The validation of MS/MS spectra of the novel peptide (AVGVDCSAPEPR) identified in mouse lens proteome shown along with a similar fragmentation pattern observed from the corresponding synthetic peptide. **Note:** The terms “peptide identified from sample” and “synthetic peptide” are described in Supplementary Figure 1.


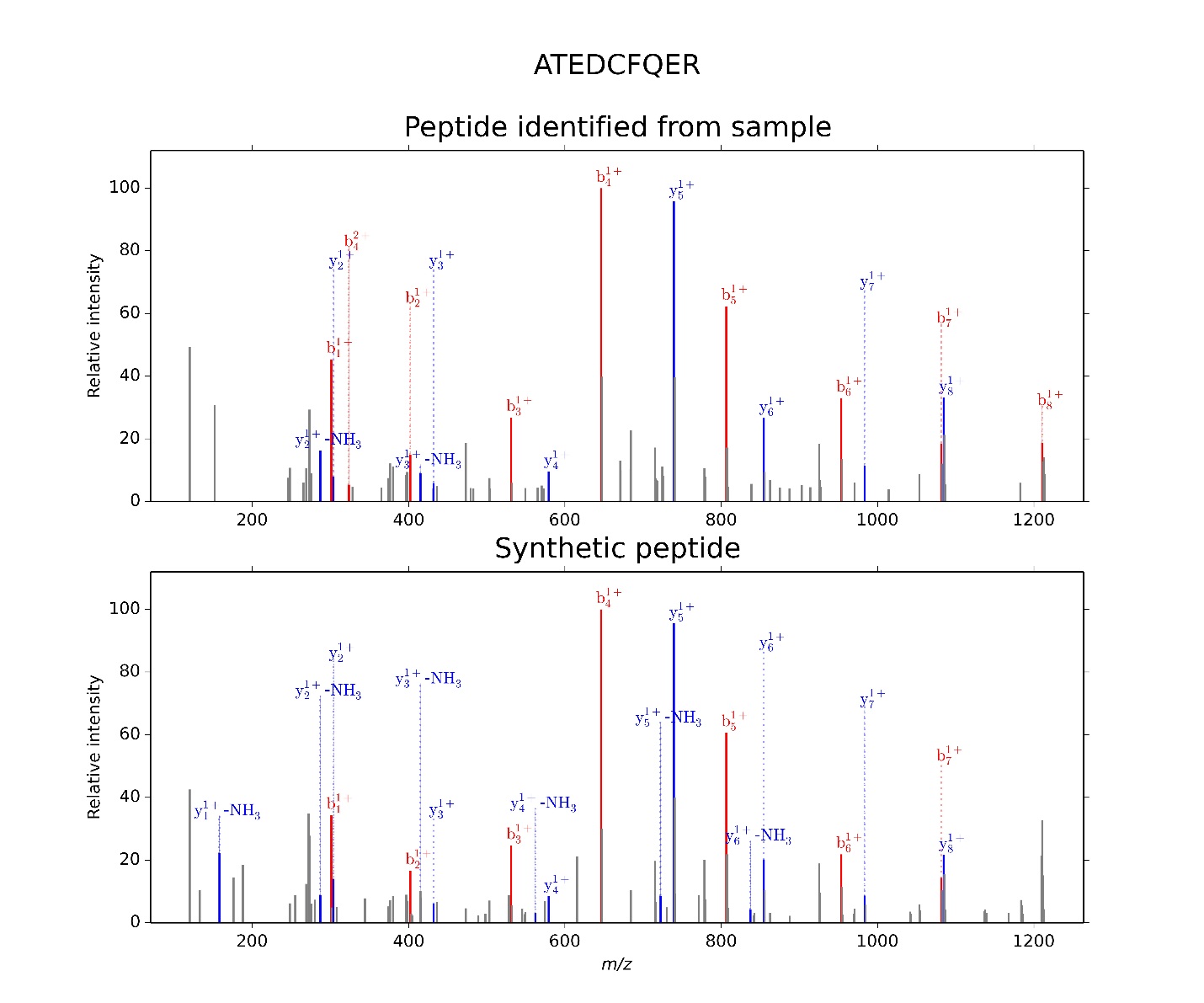


**Supplementary Figure 13.** The validation of MS/MS spectra of the novel peptide (ATEDCFQER) identified in mouse lens proteome shown along with a similar fragmentation pattern observed from the corresponding synthetic peptide. **Note:** The terms “peptide identified from sample” and “synthetic peptide” are described in Supplementary Figure 1.


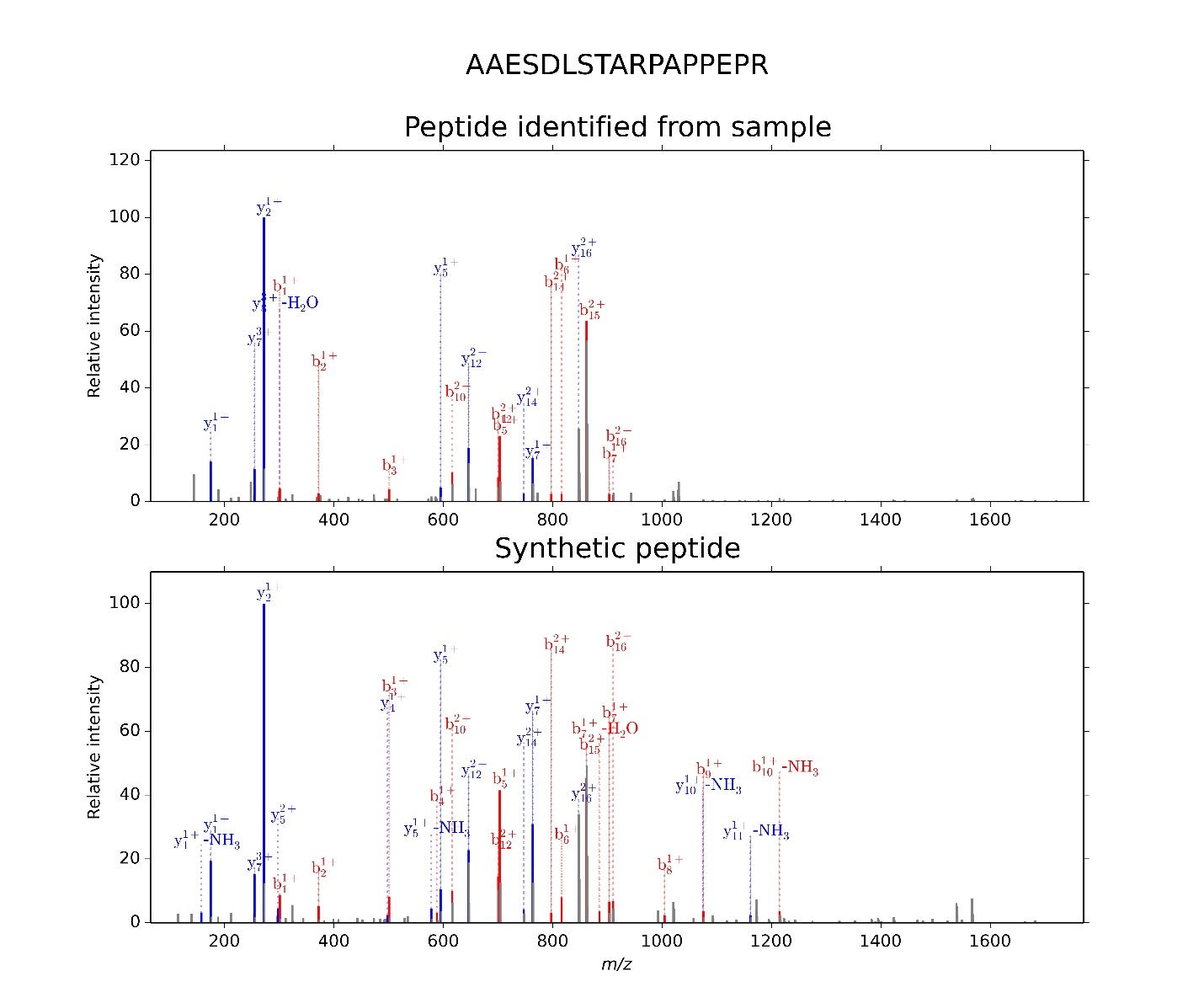


**Supplementary Figure 14.** The validation of MS/MS spectra of the novel peptide (AAESDLSTARPAPPEPR) identified in mouse lens proteome shown along with a similar fragmentation pattern observed from the corresponding synthetic peptide. **Note:** The terms “peptide identified from sample” and “synthetic peptide” are described in Supplementary Figure 1.


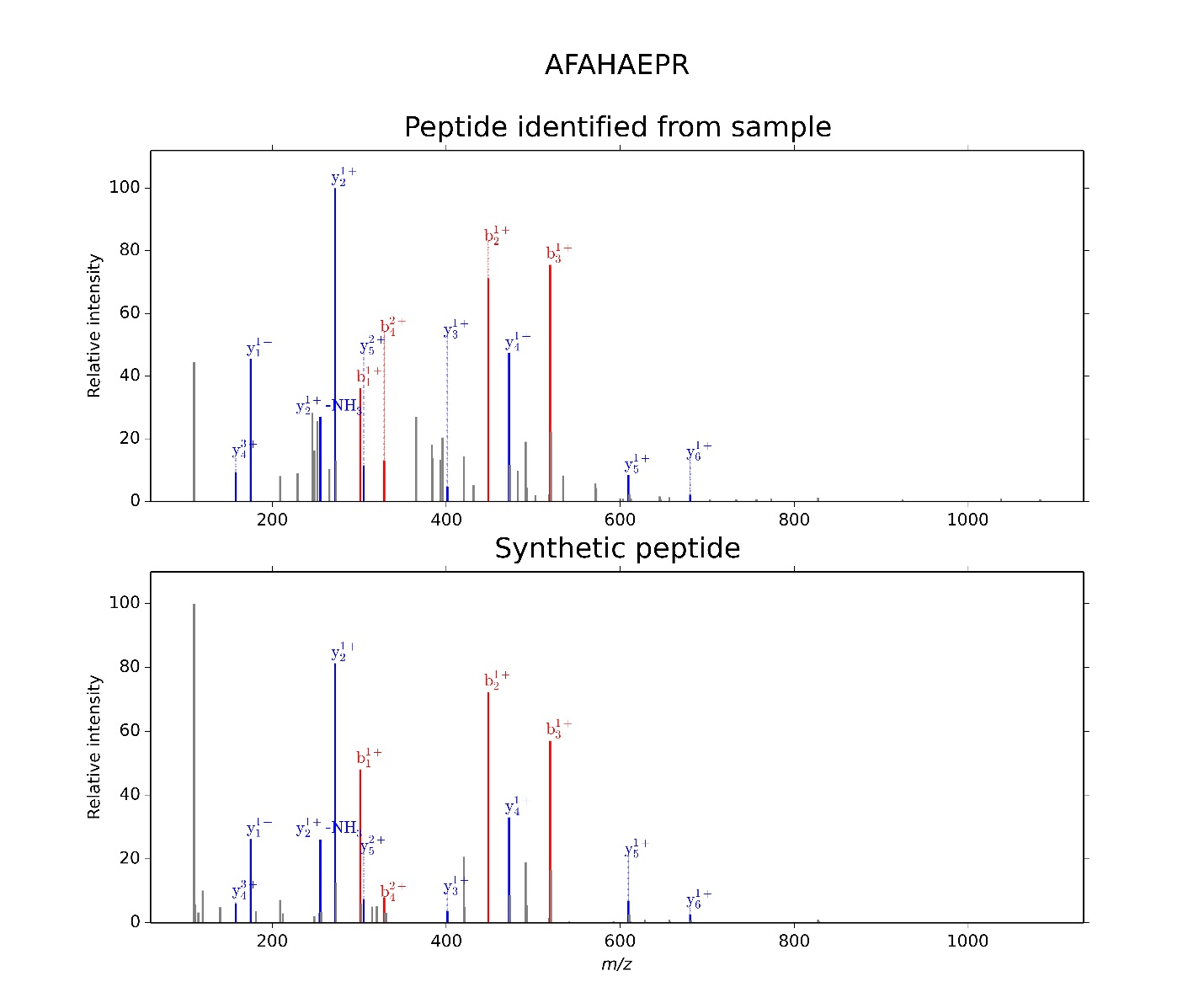


**Supplementary Figure 15.** The validation of MS/MS spectra of the novel peptide (AFAHAEPR) identified in mouse lens proteome shown along with a similar fragmentation pattern observed from the corresponding synthetic peptide. **Note:** The terms “peptide identified from sample” and “synthetic peptide” are described in Supplementary Figure 1.

**
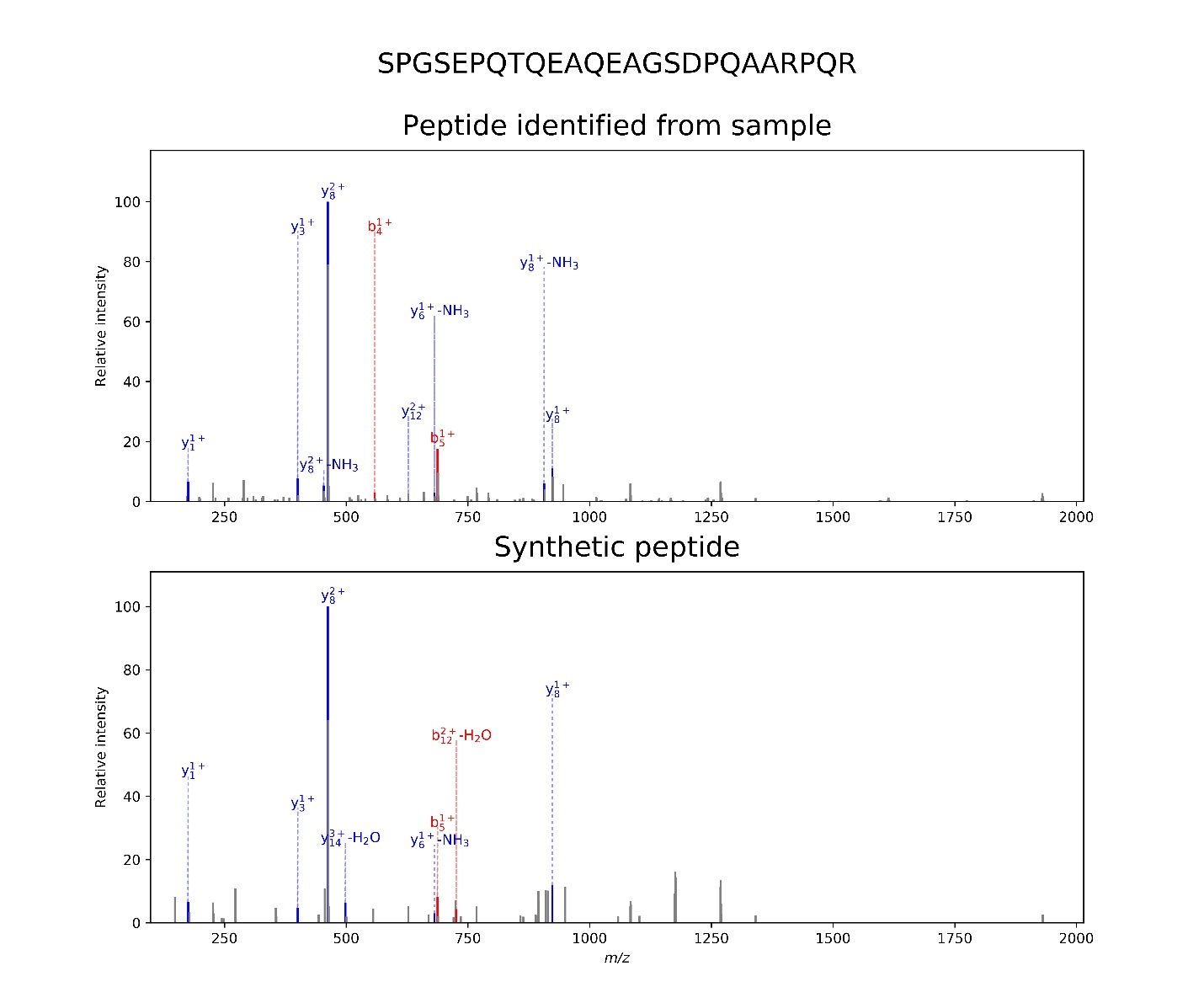
**

**Supplementary Figure 16.** The validation of MS/MS spectra of the novel peptide (SPGSEPQTQEAQEAGSDPQAARPQR) identified in mouse lens proteome shown along with a similar fragmentation pattern observed from the corresponding synthetic peptide. **Note:** The terms “peptide identified from sample” and “synthetic peptide” are described in Supplementary Figure 1.

**
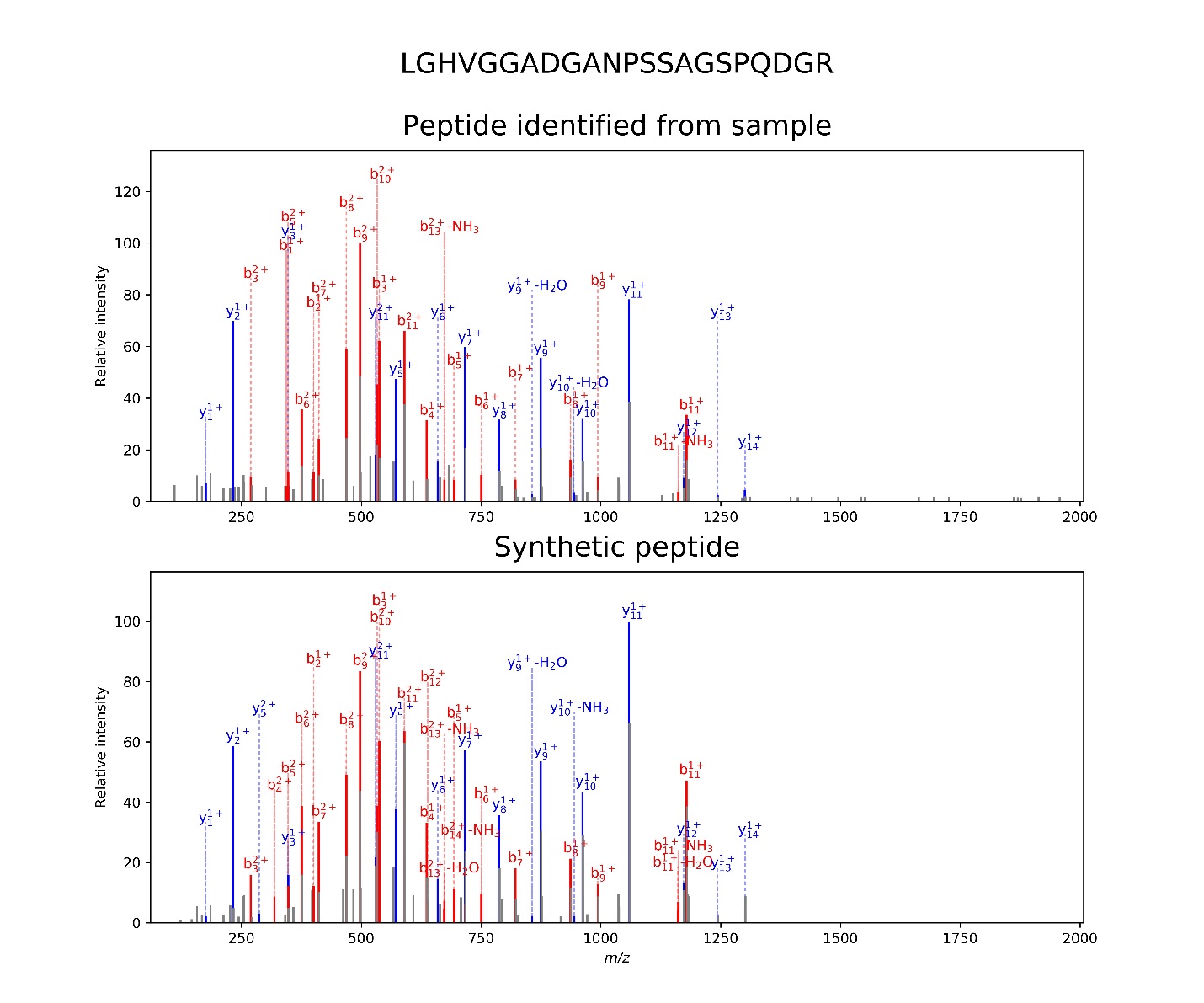
**

**Supplementary Figure 17.** The validation of MS/MS spectra of the novel peptide (LGHVGGADGANPSSAGSPQDGR) identified in mouse lens proteome shown along with a similar fragmentation pattern observed from the corresponding synthetic peptide. **Note:** The terms “peptide identified from sample” and “synthetic peptide” are described in Supplementary Figure 1.

**
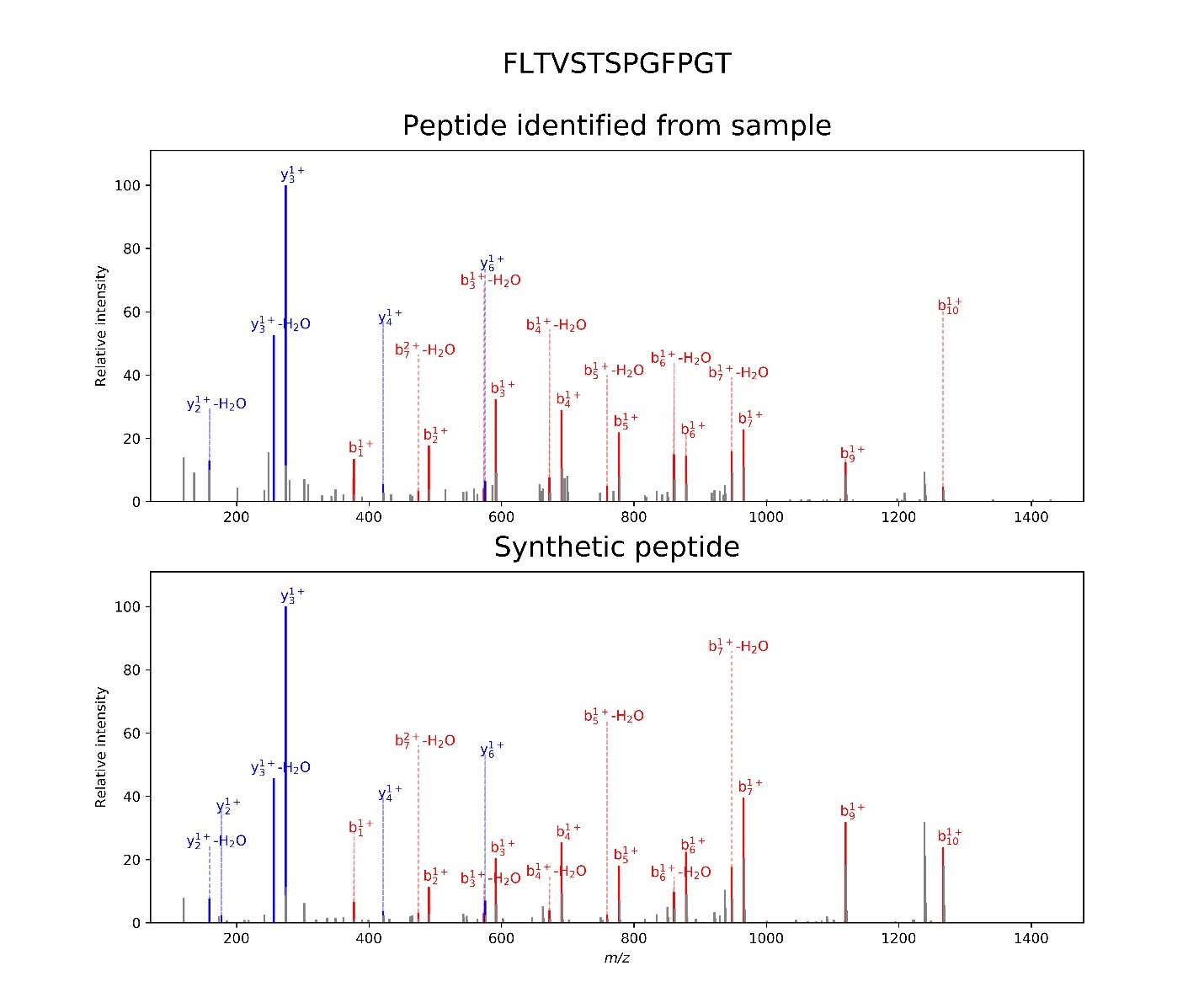
Supplementary Figure 18.** The validation of MS/MS spectra of the novel peptide (FLTVSTSPGFPGT) identified in mouse lens proteome shown along with a similar fragmentation pattern observed from the corresponding synthetic peptide. **Note:** The terms “peptide identified from sample” and “synthetic peptide” are described in Supplementary Figure 1.

**
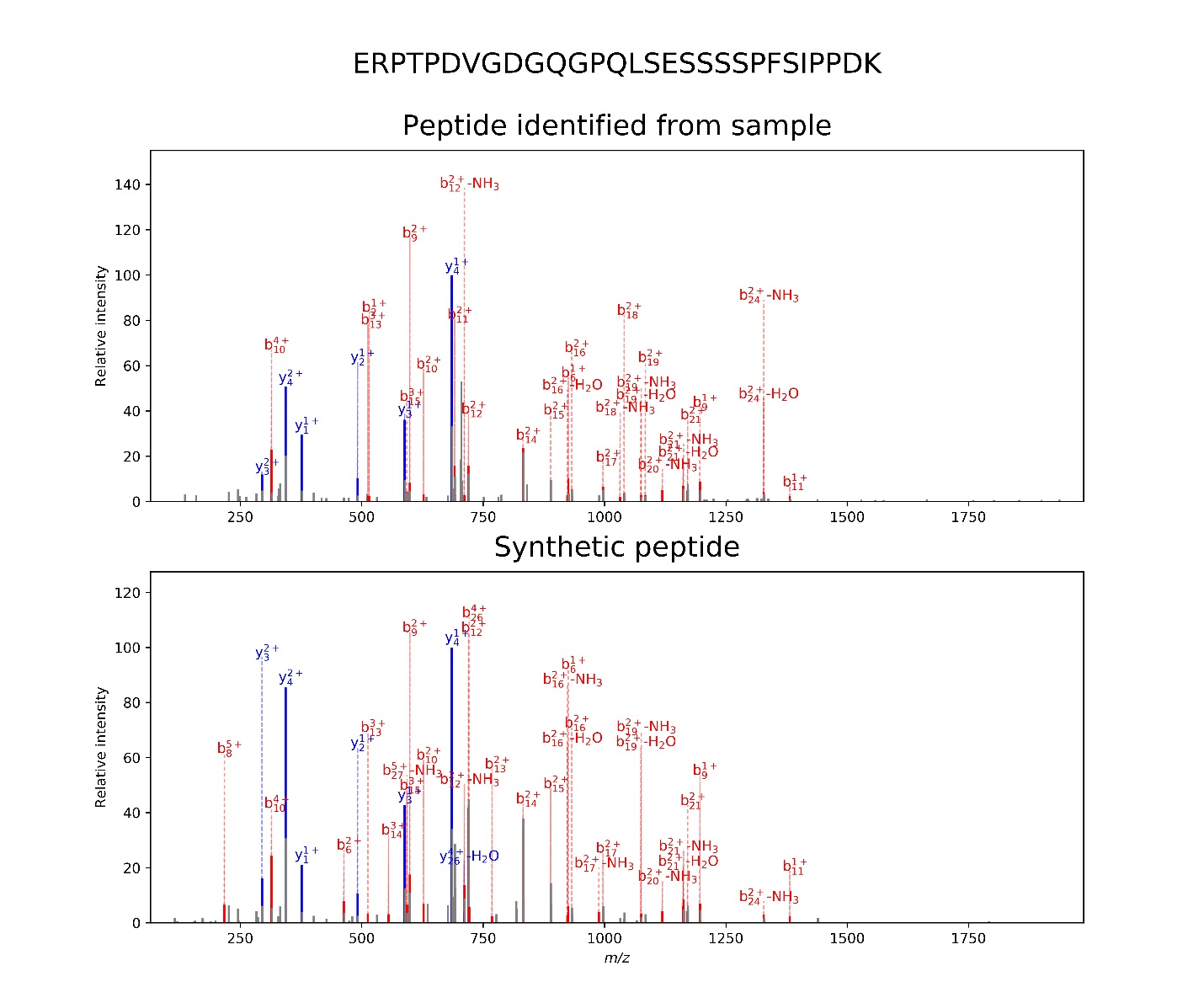
Supplementary Figure 19.** The validation of MS/MS spectra of the novel peptide (ERPTPDVGDGQGPQLSESSSSPFSIPPDK) identified in mouse lens proteome shown along with a similar fragmentation pattern observed from the corresponding synthetic peptide. **Note:** The terms “peptide identified from sample” and “synthetic peptide” are described in Supplementary Figure 1.

**
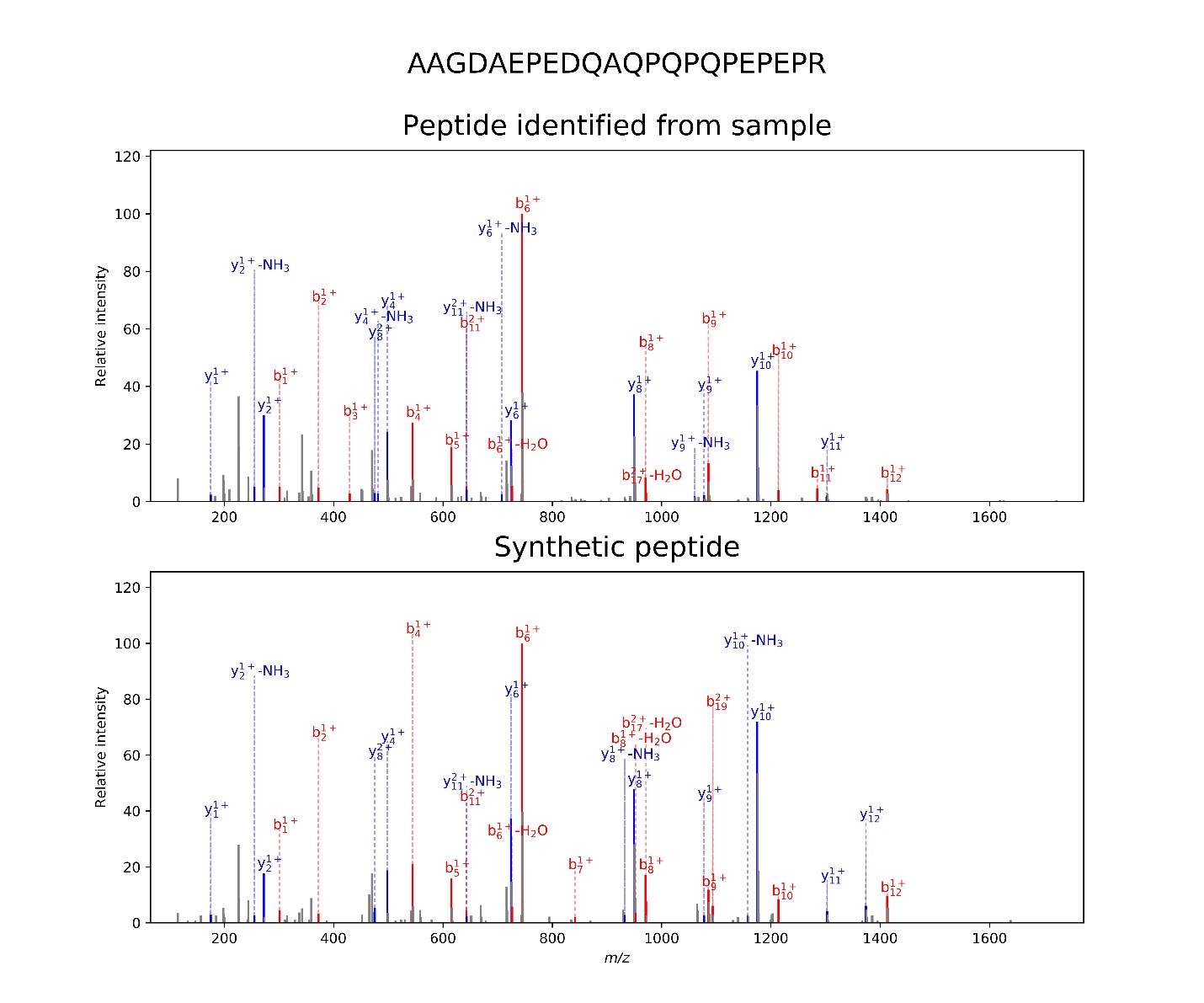
**

**Supplementary Figure 20.** The validation of MS/MS spectra of the novel peptide (AAGDAEPEDQAQPQPQPEPEPR) identified in mouse lens proteome shown along with a similar fragmentation pattern observed from the corresponding synthetic peptide. **Note:** The terms “peptide identified from sample” and “synthetic peptide” are described in Supplementary Figure 1.

**Supplementary Table 1:** Annotated transcripts identified in developing mouse lens. The HISAT2 was used for the alignment of mRNA-Seq data followed by the StringTie algorithm for the annotation and the normalization of transcripts expression by calculating the transcripts per million (TPM) value for each transcript. A cut-off value of ≥1.0 TPM was established as expression threshold for each transcript. **Note:** E15 and E18 are embryonic days 15, and 18, and P0, P3, P6, and P9 are postnatal days 0, 3, 6, and 9, respectively.

**Supplementary Table 2:** Novel transcripts identified in developing mouse lens. The HISAT2 was used for the alignment of mRNA-Seq data followed by the StringTie algorithm for the annotation and the normalization of transcripts expression by calculating the transcripts per million (TPM) value for each transcript. A cut-off value of ≥1.0 TPM was established as expression threshold for each transcript. **Note:** E15 and E18 are embryonic days 15, and 18, and P0, P3, P6, and P9 are postnatal days 0, 3, 6, and 9, respectively.

**Supplementary Table 3:** Semi-novel transcripts identified in developing mouse lens. The HISAT2 was used for the alignment of mRNA-Seq data followed by the StringTie algorithm for the annotation and the normalization of transcripts expression by calculating the transcripts per million (TPM) value for each transcript. A cut-off value of ≥1.0 TPM was established as expression threshold for each transcript. **Note:** E15 and E18 are embryonic days 15, and 18, and P0, P3, P6, and P9 are postnatal days 0, 3, 6, and 9, respectively.

**Supplementary Table 4:** Fusion genes identified in developing mouse lens. The JAFFA algorithm was used to identify the fusion genes in mouse lens. **Note:** E15 and E18 are embryonic days 15, and 18, and P0, P3, P6, and P9 are postnatal days 0, 3, 6, and 9, respectively.

**Supplementary Table 5:** Molecular function, biological process, and cellular component GO terms associated with novel fusion genes identified in developing mouse lens.

**Supplementary Table 6:** Mammalian phenotype (MP) terms associated with novel fusion genes identified in developing mouse lens.

**Supplementary Table 7:** Novel exon-skipping (ES) splicing events identified in developing mouse lens. The rMATS pipeline was used to characterize the known and novel splicing events and the p-value and false discovery rate (FDR) was calculated for each ES event among different developmental time points. **Note:** E15 and E18 are embryonic days 15, and 18, and P0, P3, P6, and P9 are postnatal days 0, 3, 6, and 9, respectively.

**Supplementary Table 8:** Novel alternative 3′ splice site (A3SS) events identified in developing mouse lens. The rMATS pipeline was used to characterize the known and novel splicing events and the p-value and false discovery rate (FDR) was calculated for each A3SS event among different developmental time points. **Note:** E15 and E18 are embryonic days 15, and 18, and P0, P3, P6, and P9 are postnatal days 0, 3, 6, and 9, respectively.

**Supplementary Table 9:** Novel alternative 5′ splice site (A5SS) events identified in developing mouse lens. The rMATS pipeline was used to characterize the known and novel splicing events and the p-value and false discovery rate (FDR) was calculated for each A5SS event among different developmental time points. **Note:** E15 and E18 are embryonic days 15, and 18, and P0, P3, P6, and P9 are postnatal days 0, 3, 6, and 9, respectively.

**Supplementary Table 10:** Novel mutually exclusive exon (MXE) events identified in developing mouse lens. The rMATS pipeline was used to characterize the known and novel splicing events and the p-value and false discovery rate (FDR) was calculated for each MXE event among different developmental time points. **Note:** E15 and E18 are embryonic days 15, and 18, and P0, P3, P6, and P9 are postnatal days 0, 3, 6, and 9, respectively.

**Supplementary Table 11:** Novel intron retention (IR) events identified in mouse lens. The rMATS pipeline was used to characterize the known and novel splicing events and the p-value and false discovery rate (FDR) was calculated for each IR event among different developmental time points. **Note:** E15 and E18 are embryonic days 15, and 18, and P0, P3, P6, and P9 are postnatal days 0, 3, 6, and 9, respectively.

**Supplementary Table 12:** Novel transcripts identified in developing mouse lens proteome through integration of OMIC (transcriptome and proteome) datasets that were subsequently validated through matching MS/MS spectra of synthetic peptides. **Note:** E15 and E18 are embryonic days 15, and 18, and P0, P3, P6, and P9 are postnatal days 0, 3, 6, and 9, respectively.
